# Indirect Striatal Projection Neurons Drive a D2 Receptor‐Dependent Pathway to Dyskinesia and Dystonia

**DOI:** 10.1002/mds.70299

**Published:** 2026-04-20

**Authors:** Laura Andreoli, Teodor Nyman, Elena Espa, Johan Jakobsson, Osama F. Elabi, Maria Angela Cenci

**Affiliations:** ^1^ Basal Ganglia Pathophysiology Group, Department of Experimental Medical Science Lund University Lund Sweden; ^2^ Molecular Neurogenetics Laboratory, Department of Experimental Medical Science Lund University Lund Sweden

**Keywords:** dopamine agonists, levodopa‐induced dyskinesias, Parkinson's disease, pathophysiology, rodent models

## Abstract

**Background:**

L‐DOPA‐induced dyskinesia is attributed to opposite activity changes mediated by D1 and D2 dopamine receptors in the two striatal output pathways. Whereas the causal role of direct‐pathway D1 receptors is well established, the specific involvement of indirect‐pathway D2 receptors in dopaminergic dyskinesias has remained elusive.

**Objectives:**

We used conditional knockout approaches in mice to determine whether indirect‐pathway D2 receptors causally contribute to dyskinetic and dystonic responses to dopaminergic agents.

**Methods:**

Studies were conducted in mice with unilateral 6‐hydroxydopamine lesions of the nigrostriatal pathway receiving subchronic treatments with L‐DOPA or D2/D1‐selective agonists. A conditional knockout of indirect‐pathway D2 receptors was produced either through the entire striatum (double‐transgenic *Adora2a*‐Cre/*Drd2* ^loxP/loxP^ mice) or selectively in the dopamine‐denervated dorsal striatum (proenkephalin promoter‐driven Cre vector delivery to *Drd2* ^loxP/loxP^ mice).

**Results:**

The severity of L‐DOPA‐induced abnormal involuntary movements and dystonia was halved in both knockout models compared with control mice, whereas the treatment effect on normal motor behaviors was either not reduced or improved. All dyskinetic and dystonic features induced by the D2‐selective receptor agonist sumanirole were completely abolished, whereas those induced by the D1‐class agonist SKF38393 were largely unaffected. Using phosphorylated ribosomal protein S6 as an activity marker, we detected a treatment‐induced recruitment of prototypical parvalbumin‐positive neurons in the external globus pallidus (a target of indirect‐pathway projections). This effect was inhibited in both knockout models.

**Conclusions:**

We provide experimental evidence that indirect‐pathway D2 receptors significantly contribute to the expression of dyskinesia during L‐DOPA treatment and mediate D2 agonist‐dependent dystonic features. © 2026 The Author(s). *Movement Disorders* published by Wiley Periodicals LLC on behalf of International Parkinson and Movement Disorder Society.

## Graphical abstract

D2 receptor ablation in indirect‐pathway striatal neurons reduces or abolishes dyskinetic and dystonic behaviors induced by L‐DOPA or D2 receptor agonists, respectively. Contralateral turning is reduced, while forward locomotion is increased. These effects are associated with modulation of neuronal activity in dorsal striatum and external globus pallidus. 'Overactive neurons' were identified based on their upregulated expression of phosphorylated ribosomal protein S6.

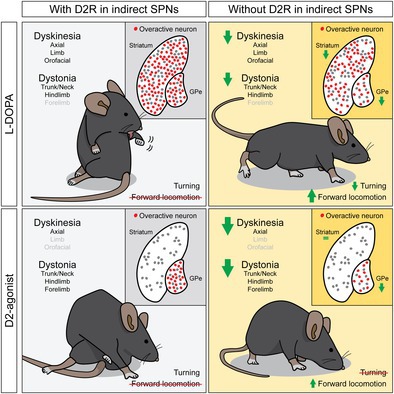

Dopamine (DA) regulates movement via DA receptors D1 and D2, which are highly expressed in the striatum, although also present in other brain regions.^1^ In Parkinson's disease, the degeneration of nigrostriatal DA projections results in a deficient activation of both receptor types, leading to poverty and slowness of movement (hypokinesia and bradykinesia). These motor symptoms are treated using either L‐3,4‐dihydroxyphenylalanine (L‐DOPA) or DA agonists having a predominant action on D2 class receptors. Despite its efficacy, dopaminergic pharmacotherapy leads to the development of abnormal involuntary movements, referred to as L‐DOPA‐induced dyskinesia (LID), in a large number of patients.^2^, ^3^ The relative contribution of D1 and D2 receptors to parkinsonian and dyskinetic motor features remains a matter of scientific debate. Both receptor types are implicated in the genesis of normal movements,^4^ and blocking one or the other type results in nominally the same hypo‐bradykinetic phenotype in rodents.^5^ However, the emergence of LID has been specifically linked to D1 receptor stimulation on direct‐pathway striatal projection neurons (dSPNs),^6^, ^7^ while the role of D2 receptors (D2Rs) has been the subject of discrepant experimental results. Comparing mice with a global genetic knockout of either D1Rs or D2Rs, a seminal study reported that ablating D1Rs abolishes LID, whereas D2R ablation does not have any significant effect.^6^ Other studies have instead reported LID augmentation in two different mouse models of D2R ablation, that is, mice with a global knockout of the D2R‐long isoform^8^ and mice with conditional D2R ablation in striatal projection neurons of the indirect pathway (iSPNs).^9^ The finding of increased LID severity in D2R knockout animals^8^, ^9^ conflicts with a substantial pharmacological literature showing that LID is markedly reduced by D2R antagonists^10^, ^11^, ^12^ and aggravated by D2R agonists.^13^, ^14^, ^15^, ^16^, ^17^ Antidyskinetic effects of D2R antagonists and prodyskinetic effects of D2R agonists have been reported also using intrastriatal drug infusions.^16^, ^18^

In the present study, we have addressed the role of indirect‐pathway D2R in LID using two different mouse models of conditional iSPN‐specific D2R ablation: (1) a pan‐striatal model generated by crossing Drd2^loxP/loxP^ mice with an Adora2a‐Cre transgenic driver line, thereby eliminating D2Rs from iSPNs across the striatum^19^, ^20^ and (2) a focal model obtained by delivering a proenkephalin (PENK)‐promoter‐driven Cre vector to the dorsolateral striatum of Drd2^loxP/loxP^ mice. Animals were treated with L‐DOPA or D1/D2‐specific agonists to induce abnormal involuntary movements and postures, which were quantified using articulate rating scales.

## 1 Methods

Detailed descriptions are provided in the Supplementary Methods.

### 1.1 Animals and Viral Vectors

The study was performed in C57BL/6 adult mice of both sexes. To produce conditional iSPN‐D2R knockout (KO) models we used transgenic Drd2^loxP/loxP^ mice (JAX020631, Jackson Labs). In the bilateral KO model, these animals were crossed with Adora2a‐Cre mice KG139Gsat/Mmucd (GENSAT) (herein A2a‐Cre mice) as detailed in Lemos et al.^20^ To generate the unilateral iSPN‐D2R KO model, we produced a vector coding for Cre under the iSPN‐specific^21^ PENK promoter. A flip‐excision (flex) vector coding for green fluorescent protein (GFP) (AAV‐flex‐GFP) was used to verify the cellular specificity of Cre activity in both KO models. All viral vectors were AAV2/5 pseudotyped and produced in our core facility. Vectors were injected into the right dorsolateral striatum (vector titer ~10^13^ genome copies/mL; 1 μL/injection) as described in Alcacer et al.^22^

### 1.2 Dopamine‐Denervating Lesions and Drug Treatments

Nigrostriatal DA lesions were produced by injecting 6‐hydroxydopamine (6‐OHDA) into the right medial forebrain bundle as detailed in Andreoli et al.^23^ and Francardo et al.^24^ All lesioned animals included in the study had > 85% striatal loss of tyrosine hydroxylase ipsilaterally to the 6‐OHDA infusion. Dopaminergic drugs were administered using previously characterized subchronic treatment regimens and doses.^23^ L‐DOPA methyl ester, sumanirole, and SKF38393 were dissolved in saline and administered intraperitoneally at doses of 6, 4, and 3 mg/kg/day, respectively. Each drug was administered for five consecutive days, leaving one drug‐free week between consecutive treatments. To minimize potential priming effects of one treatment relative to the next one,^25^, ^26^, ^27^ drugs were given in the following order: sumanirole, SKF38393, L‐DOPA.

### 1.3 Ratings of Treatment‐Induced Dyskinesia and Dystonia

Abnormal involuntary movements (AIMs) and dystonic features were assessed on days 1, 3, and 5 of each drug treatment period. Animals were observed individually 1 min every 20th min for 3 hr following drug administration. Severity ratings were based on the proportion of observation time during which an AIM or dystonic feature was present.^22^, ^23^, ^24^ The sum of axial, limb, and orofacial AIMs is the rodent equivalent of LID,^28^ whereas ‘locomotive scores’ (locomotor actions with contralateral side bias) provide a generic index of motor activation on treatment.^29^, ^30^ For dystonia ratings, we considered six topographic items, namely trunk and neck (tr/ne), tail, hindlimbs and forelimbs contralateral and ipsilateral to the lesion.^23^ Scores from hindlimbs and forelimbs of both sides were summed (herein, ‘HL and FL dystonia’).

### 1.4 Behavioral Parcellation

Videos recorded at peak dyskinesia severity (40th and 60th min after drug injection) were analyzed using the event‐recording freeware JWatcher V1.0 (https://www.jwatcher.ucla.edu/).^31^ We quantified the most prevalent behavioral categories, namely (i) Contralateral turn, turning of head and body towards the side contralateral to the lesion; (ii) Forward locomotion, locomotion with a straight body; (iii) Grooming, grooming sequence with bilateral involvement of the forelimbs; and (iv) Rearing, vertical motion with forepaw placing on the cage walls. Results were expressed as the proportion of active time spent on each behavioral category.

### 1.5 Immunohistochemistry, Image Analysis

Animals were deeply anesthetized 30 min after the last drug/vehicle injection and fixated by transcardial perfusion with 4% buffered paraformaldehyde. Brains were vibratome‐cut at 30 μm thickness and processed for immunohistochemistry as described in Supplementary Methods. Image analysis was performed using open‐source image processing programs (Fiji and QuPath). Neuronal bodies positive for phosphorylated S6 were counted setting the same background threshold in all sample areas (0.18 mm^2^/sample area, nine areas per region per animal).

### 1.6 Statistical Analyses

Output variables were compared between mouse genotypes using two‐factor or repeated‐measures analysis of variance (ANOVA), and pairwise post‐hoc tests corrected for multiple comparisons (Tukey's or Bonferroni's). Sums of AIMs, locomotive, or dystonia scores were compared using Kruskal–Wallis or Mann–Whitney test and expressed as median and range. All other data are expressed as mean ± standard error of the mean (SEM). Detailed statistical data are provided in Table S1. The level of significance was set at *P* < 0.05.

### 1.7 Data Sharing

All data are available from the corresponding author upon reasonable request.

## 2 Results

### 2.1 Generation and Validation of the Two Conditional iSPN‐D2R Knockout Models

**FIG. 1:**
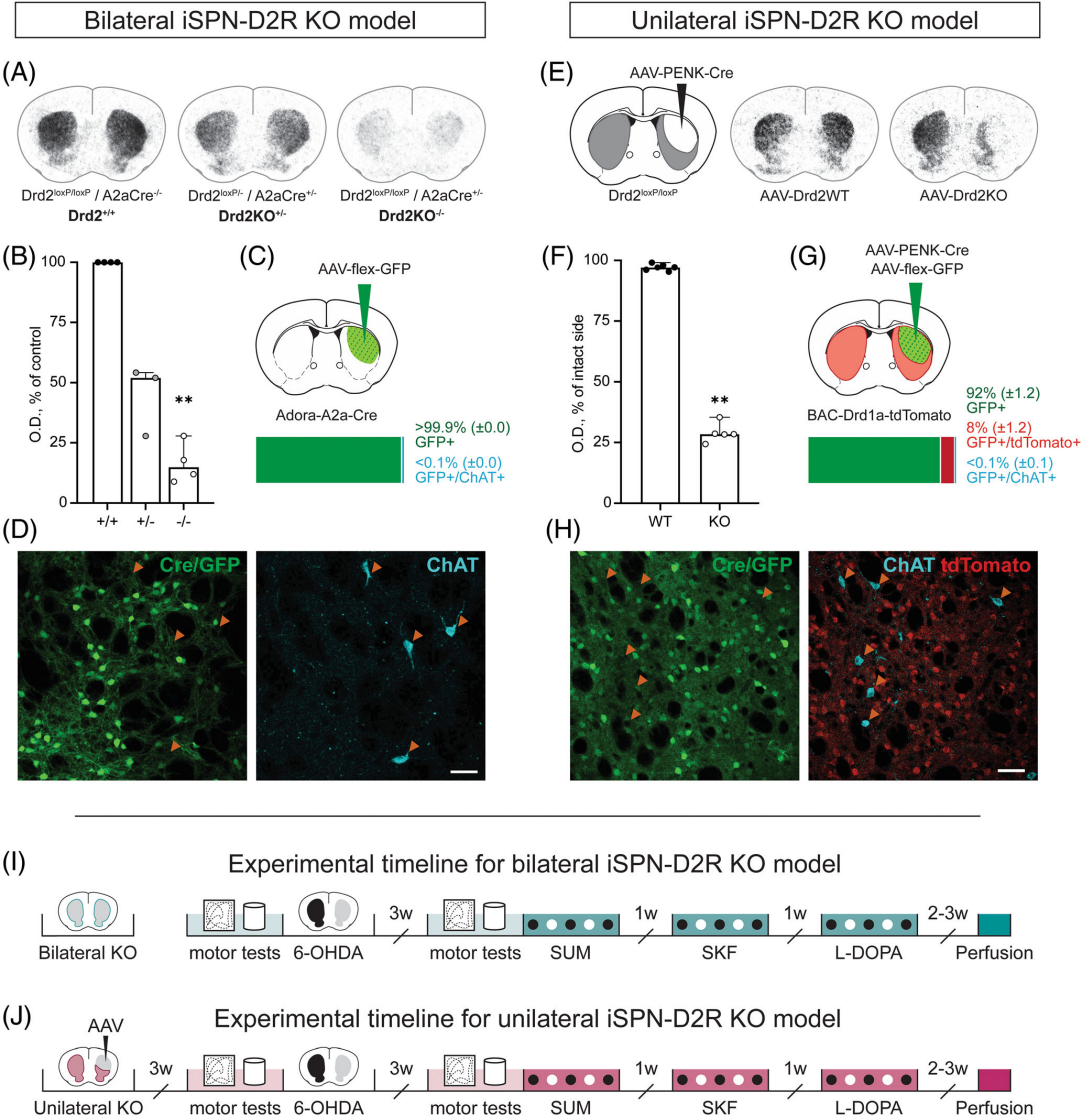
Validation of transgenic and viral‐mediated indirect‐pathway striatal projection neuron (iSPN)‐D2R deletion. (A) Bilateral knockout (KO) model. Section autoradiographs show [^3^H]raclopride binding densities in the three genotypes used. (B) The corresponding optical density (OD) measurements (mean + SEM; **P < 0.01 vs. Drd2^+/+^). (C) Validation of Cre‐mediated recombination using the AAV‐flex‐GFP vector. Less than 0.1% of striatal green fluorescent protein (GFP)‐positive neurons are choline acetyltransferase (ChAT)‐positive (orange arrows) (data from n = 4 mice and >1000 cells/three sections/mouse). (D) Confocal photomicrographs of GFP‐ChAT immunostained sections (scale bar: 100 μm). (E) Unilateral KO model. Section autoradiographs show [^3^H]raclopride binding densities in AAV‐Drd2WT and AAV‐Drd2KO mice. (F) The corresponding OD data (**P < 0.01 vs. WT). (G) Validation of Cre‐mediated recombination. The AAV‐flex‐GFP and AAV‐PENK‐Cre vectors were co‐injected in mice expressing a red fluorescence protein in direct‐pathway striatal projection neurons (dSPNs) (BAC‐Drd1a‐tdTomato). Cell counts show that 92% of GFP‐positive neurons are tdTomato‐negative and <0.1% stain positively for ChAT (data from n = 5 mice and >500 cells/three sections/mouse). (H) Confocal photomicrographs of striatal sections with triple GFP‐ChAT‐TdTomato immunofluorescence; orange arrows indicate ChAT‐positive neurons (scale bar: 100 μm). (I, J). Experimental timeline for the bilateral iSPN‐D2R KO model (I) and for the unilateral iSPN‐D2R KO model (J). Drug‐free motor tests were conducted before and after the lesion to assess the impact of striatal iSPN‐D2R deletion. Thereafter, animals with 6‐OHDA lesions received sequential subchronic daily treatment with sumanirole (SUM, 4 mg/kg), SKF38393 (SKF, 3 mg/kg), and L‐DOPA (6 mg/kg). Animals were examined for dyskinesia, dystonia, and other behaviors every other day (black dots). At least 2 weeks after completing the drug treatment protocol, mice received a last injection and were perfusion‐fixated for brain tissue analyses. [Color figure can be viewed at wileyonlinelibrary.com]

The bilateral iSPN‐D2R KO model was obtained by crossing Drd2^loxP/loxP^ and A2a‐Cre mice as detailed in Dobbs et al.^19^ and Lemos et al.^20^ The mating strategy produced three progenies with different D2R allele titrations, that is: (i) mice negative for Cre and therefore having normal D2R expression (hereinafter Drd2^+/+^); (ii) mice heterozygous for both the Drd2^loxP/loxP^ and the A2a‐Cre alleles, therefore only partially iSPN D2R‐depleted (Drd2KO^+/−^); and (iii) mice homozygous for Drd2^loxP/loxP^ and heterozygous for the A2a‐Cre allele, therefore totally lacking iSPN D2Rs (Drd2KO^−/−^).^19^, ^20^ The striatal D2R ablation was verified in a group of unlesioned animals using D2R autoradiography. The average striatal reduction in D2R radioligand binding density was approximately 40% and 88% in the hemizygous Drd2KO^+/−^ and the homozygous Drd2KO^−/−^ groups, respectively (Fig. 1A,B), well reflecting the extent of Drd2 mRNA depletion previously reported in the same mouse models.^20^ Because D2R is also expressed in cholinergic interneurons, we verified the cellular selectivity of Cre recombination in striatal sections transduced with the AAV‐flex‐GFP vector and immunostained for choline acetyltransferase (ChAT). Quantitative cell counts on confocal images from Drd2KO^−/−^ mice confirmed that over 99.9% of the GFP‐positive cells were ChAT‐negative, and only 0.08% ChAT cells were GFP‐positive (Fig. 1C,D).

A conditional iSPN‐D2R ablation confined to the DA‐denervated dorsal striatum (‘unilateral iSPN‐D2RKO model’) was obtained by intrastriatal injection of the AAV‐PENK‐Cre vector in homozygous Drd2^loxP/loxP^ mice. These animals displayed a consistent 75% loss of striatal D2R radioligand binding density (Fig. 1E,F). The cellular specificity of virally‐mediated Cre recombination was verified by co‐injecting the AAV‐PENK‐Cre and AAV‐flex‐GFP vectors in mice where dSPNs express a red fluorescent protein (‘BAC‐Drd1a‐tdTomato’; Fig. 1G). Confocal microscopy analysis showed that approximately 92% of the GFP‐positive neurons were TdTomato‐negative, 8% were TdTomato‐positive, and only 0.04% ChAT cells expressed GFP (Fig. 1G,H).

The motor phenotypes induced by the conditional iSPN‐D2R ablations before drug treatment were assessed by recording open‐field activity and limb use in the cylinder test (see Supplemental Results SI). Briefly, mice with bilateral iSPN‐D2R ablation exhibited reduced open‐field motions without asymmetries in posture or forelimb use (Fig. S1A–F'). These data are in keeping with the hypo‐bradykinetic phenotype previously reported in the same mouse model.^20^ In contrast, mice with unilateral iSPN‐D2R ablation exhibited an overall normal open‐field activity, although with a turning bias towards the D2R‐depleted side (Fig. S2A–F'). Interestingly, the unilateral iSPN‐D2R ablation resulted in a reduced use of the contralateral forelimb in the cylinder test (Fig. S2A), resembling the asymmetry in forelimb use induced by unilateral 6‐OHDA lesions.^24^ Neither the reduction in open‐field activity nor the deficit in forelimb use worsened significantly after the 6‐OHDA lesion in either KO model (see Figs S1H,K,K' and S2H,K,K'), indicating that the deletion of iSPN D2Rs occluded motor deficits caused by DA denervation.

To induce dyskinesia and dystonia, 6‐OHDA‐lesioned KO animals and their control groups received sequential subchronic treatment with a D2R agonist, a D1R agonist, and L‐DOPA (see treatment timeline in Fig. 1I,J). The evolution of dyskinesia scores over each treatment is shown in Figures S3 and S4 (see also Supplemental Results and Discussion SII). The results presented below refer to the last test session with each drug.

### 2.2 Bilateral iSPN‐D2R Ablation Ameliorates LID and Blocks D2R‐Dependent Dystonia

**FIG. 2:**
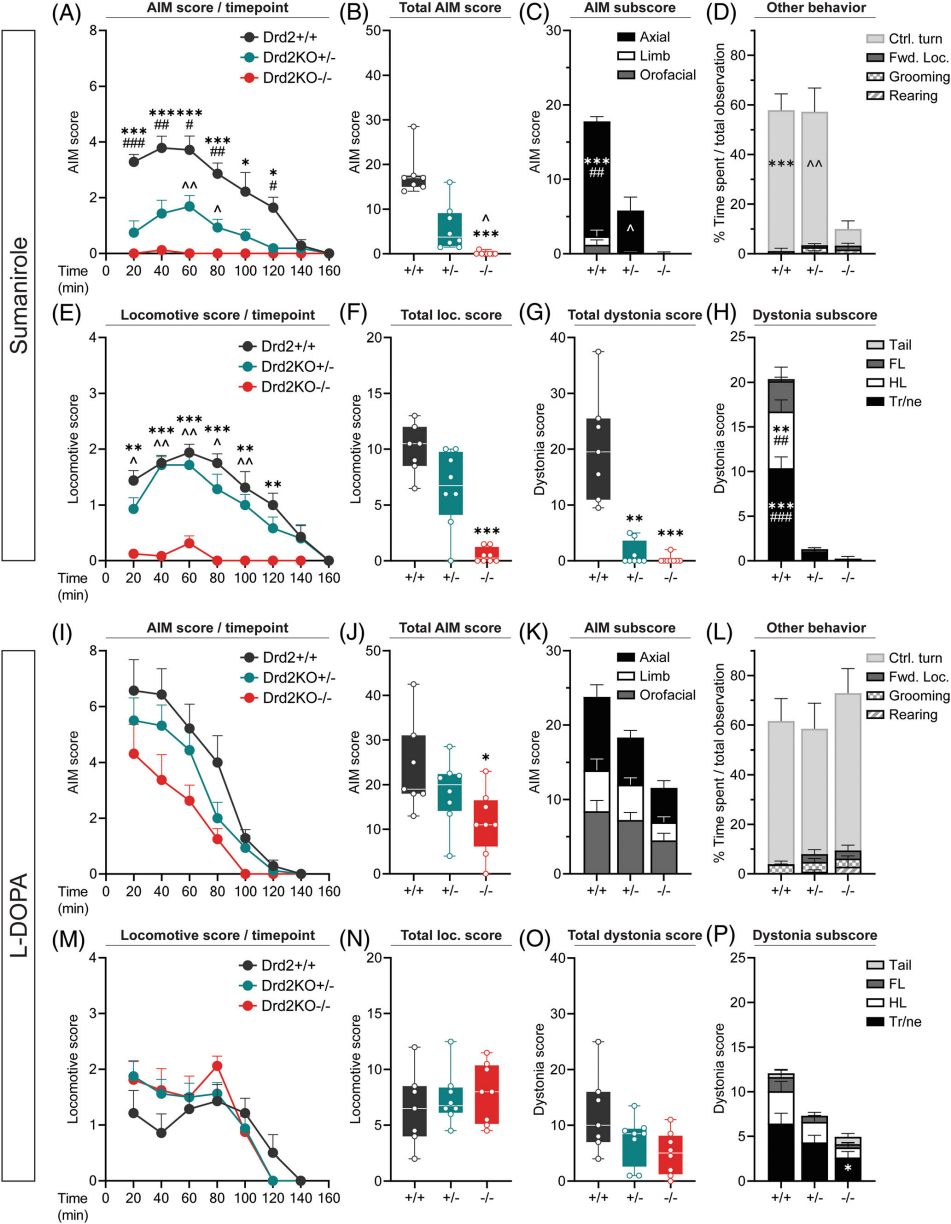
Bilateral indirect‐pathway striatal projection neuron (iSPN)D2R ablation blunts the severity of L‐DOPA‐induced dyskinesia (LID) and suppresses D2 agonist‐induced dyskinesia and dystonia. Data are from the last test session in the treatment period with sumanirole (A–H) and L‐DOPA (I–P) for mice with normal levels of iSPN D2R (Drd2^+/+^, n = 7), hemizygous iSPN‐D2R knockout (KO) mice (Drd2KO^+/−^, n = 8), and homozygous iSPN‐D2R KO mice (Drd2KO^−/−^, n = 8). (A, I) Time course of axial, limb, and orofacial abnormal involuntary movement (AIM) scores (sum per monitoring period). (B, J) Sum of axial, limb, and orofacial AIM scores in the test session. (C, K) Breakdown of the AIM scores/session into the three dyskinesia subtypes (see caption). (D, L) Behavioral parcellation analysis (videos from 40 to 60 min after drug injection) (*Fwd Loc*, forward locomotion; *Ctrl. turn*, contralateral turning). (E, M) Time course of locomotive scores. (F, N) Sum of locomotive scores in the test session. (G, O) Total dystonia scores in the same test session (sum of dystonia scores from all body regions). (H, P) Breakdown of the dystonia scores into the four topographic subtypes, represented using different bar fillings (see caption). See Table S1 for statistical data. *P < 0.05, **P < 0.01, ***P < 0.001 for Drd2^+/+^ versus Drd2^−/−^; ^#^ *P* < 0.05, ^##^ *P* < 0.01, ^###^ *P* < 0.001 for Drd2^+/+^ versus Drd2KO^+/−^; ^*P* < 0.05 for Drd2^+/−^ versus Drd2^−/−^. FL, forelimbs; HL, hindlimbs, Tr/ne, trunk and neck. [Color figure can be viewed at wileyonlinelibrary.com]

- D2R agonist treatment: We have previously reported that the D2‐selective agonist sumanirole (SUM, 4 mg/kg) induces mild–moderate dyskinesia dominated by axial AIMs, peaking in severity at 40–60 min post‐drug injection.^23^ The same pattern was observed in Drd2^+/+^ mice (Fig. 2A–C). The ablation of iSPN‐D2Rs profoundly altered this response (Fig. 2A, *P* < 0.001 for genotype effect and genotype–time interaction). Specifically, SUM‐induced dyskinesia was completely absent in Drd2KO^−/−^ mice and markedly blunted in the hemizygous Drd2KO^+/−^ group (Fig. 2A, *P* < 0.05 for Drd2KO^−/−^ vs. the other two genotypes at 20–120 min post‐injection, and for Drd2KO^+/−^ vs. Drd2^+/+^ at 60–80 min; see also Fig. 2B). In the subscore analysis, axial AIMs accounted for over 75% of the total dyskinesia score recorded from Drd2^+/+^ animals (Fig. 2C), but were markedly reduced in hemizygous Drd2KO^+/−^ mice and totally absent in the homozygous Drd2KO^−/−^ group (Fig. 2C, *P* < 0.001 for genotype effect and genotype–AIM subtype interaction; *P* < 0.01 for both Drd2KO^+/−^ and Drd2KO^−/−^ vs. Drd2^+/+^ on axial AIM scores). Locomotive scores were similarly expressed in hemizygous Drd2KO^+/−^ animals and Drd2^+/+^ controls, but hardly detectable in the homozygous Drd2KO^−/−^ mice (Fig. 2E,F, *P* < 0.001 for genotype effect and genotype–time interaction; *P* < 0.05 for Drd2KO^−/−^ versus both other groups at 20–100 min post‐SUM dosing). In the behavioral parcellation analysis, the item ‘contralateral turn’ was markedly reduced in Drd2KO^−/−^ mice but normally expressed in the hemizygous Drd2KO^+/−^ group (Fig. 2D, *P* < 0.001 for genotype effect and genotype–behavioral category interaction; *P* < 0.01 for Drd2KO^−/−^ mice versus both other groups on ‘contralateral turn’).

In a previous study using 6‐OHDA‐lesioned mice, we have found that D2R agonists induce prominent dystonic features involving trunk/neck and limb muscles.^23^ SUM‐induced dystonia conformed to this pattern in Drd2^+/+^ mice, whereas dystonic features were nearly suppressed in both the DrdKO^+/−^ and DrdKO^−/−^ groups (Fig. 2G,H). In the dystonia subscore analysis, the tr/ne and HL components were negligible in hemizygous Drd2KO^+/−^ mice and totally absent in the homozygous Drd2KO^−/−^ group (Fig. 2H; *P* < 0.001 for the effects of genotype, dystonia subtype, and genotype–subtype interaction, *P* < 0.01 for both Drd2KO^+/−^ and Drd2KO^−/−^ vs. Drd2^+/+^ group on tr/ne and HL scores).

Taken together, the above results show a lack of SUM‐induced AIMs and dystonia in Drd2KO^−/−^ mice concurring with a profound reduction in locomotive scores and contralateral turning, which are typical responses elicited by D2R agonists in hemiparkinsonian rodents.^27^, ^30^ Interestingly, hemizygous Drd2KO^+/−^ mice showed a significant attenuation of D2 agonist‐induced dyskinesia and dystonia without differing from controls on any other behavior, revealing a specific benefit of partial iSPN‐D2R deletion in reducing abnormal movements and postures.

- L‐DOPA treatment: Axial, limb, and orofacial AIM scores were approximately halved in the mice with homozygous iSPN‐D2R ablation compared with wild‐type controls (Fig. 2I, *P* < 0.05 for genotype effect, *P* = 0.093 for genotype–time interaction; Fig. 2J, *P* < 0.05 for Drd2KO^−/−^ vs. Drd2^+/+^ in the total AIM score), and all three AIM subtypes were reduced (Fig. 2K; *P* < 0.05 for genotype effect and *P* > 0.05 for genotype–AIM subtype interaction). Hemizygous Drd2KO^+/−^ mice exhibited a trend toward lower AIM scores, which did not reach statistical significance in any comparison (Fig. 2I–K). The analysis of locomotive scores did not disclose any significant difference between genotypes (Fig. 2M,N). Moreover, the behavioral parcellation analysis did not reveal any significant genotype effect (Fig. 2L, *P* > 0.05 for genotype and genotype–behavioral category interaction). Thus, the reduced dyskinesia severity observed in homozygous Drd2KO^−/−^ mice did not reflect a generally reduced motor response to L‐DOPA.

Upon examining dystonic features induced by L‐DOPA, we found significant overall effects of genotype, topographic dystonia subscore, and genotype–subscore interaction (Fig. 2O,P). These effects were driven by a markedly lower expression of trunk/neck (tr/ne) and hindlimb (HL) dystonia in Drd2KO^−/−^ mice versus Drd^+/+^ controls (Fig. 2P; *P* < 0.05 for the effects of genotype and genotype‐dystonia subscore interaction; *P* < 0.05 for Drd2KO^−/−^ vs. Drd^+/+^ groups on tr/ne scores). Although dystonic features tended to be milder in heterozygous Drd2KO^+/−^ mice, the difference from Drd2^+/+^ controls did not reach statistical significance on any item (Fig. 2P).

- D1R agonist treatment: We have previously shown that the D1R agonist SKF38393 (SKF) induces low levels of dyskinesia and dystonia, the latter mainly consisting in sustained tail dorsiflexion.^23^ This pattern was seen also in the present study (Supplemental Results SIII and Fig. S5A–H). The loss of iSPN‐D2R signaling had little or no impact on the behavioral response to SKF. However, a difference between genotypes was observed in the dystonia subscore analysis, as Drd2KO^−/−^ mice only expressed the tail component (which is D1R‐dependent^23^), lacking tr/ne and limb dystonias (Fig. S5H).

### 2.3 The Critical Pool of iSPN D2R is Located in the DA‐Denervated Dorsal Striatum

A homozygous iSPN‐D2R ablation in the DA‐denervated dorsal striatum was obtained by injecting the AAV‐PENK‐Cre vector in Drd2^loxP/loxP^ mice (‘AAV‐Drd2KO group’). The vector was also injected into wild‐type littermates to obtain a control group (‘AAV‐Drd2WT’).

**FIG. 3:**
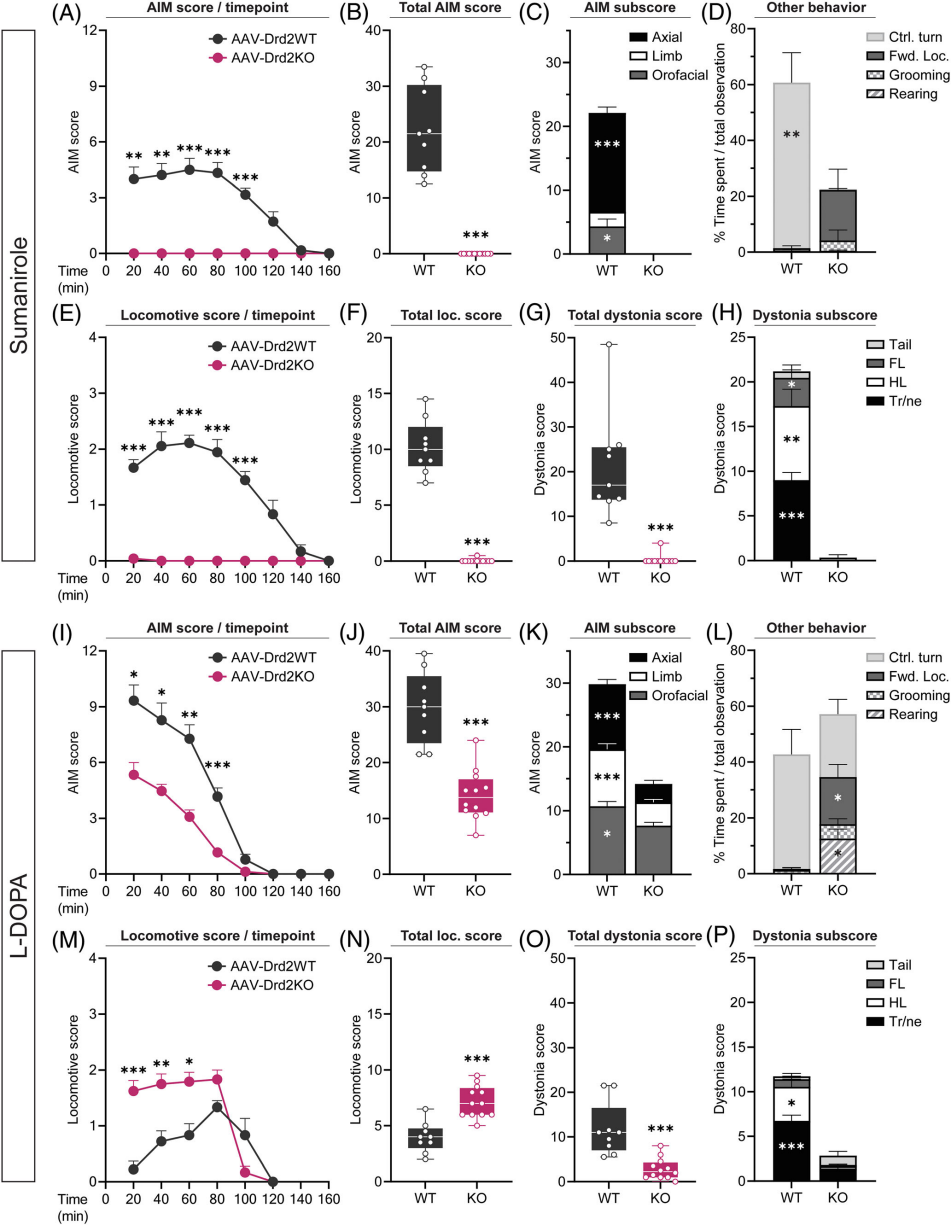
Indirect‐pathway striatal projection neuron (iSPN)D2R ablation in the dopamine (DA)‐denervated striatum strongly improves L‐DOPA‐induced dyskinesia (LID) and suppresses D2 agonist‐induced dyskinesia and dystonia. Data are from the last test session in the treatment period with sumanirole (A–H) and L‐DOPA (I–P) for mice with normal levels of iSPN D2R (AAV‐Drd2WT; n = 9) and animals with unilateral iSPN‐D2R ablation (AAV‐Drd2KO; n = 12), both injected with the AAV‐PENK‐Cre vector in the dorsolateral striatum ipsilaterally to the 6‐hydroxydopamine (6‐OHDA) lesion. (A, I) Time course of axial, limb, and orofacial abnormal involuntary movement (AIM) scores (sum per monitoring period). (B, J) Sum of axial, limb, and orofacial AIM scores in the test session. (C, K) Breakdown of the AIM scores/session into the three dyskinesia subtypes (see caption). (D, L) Behavioral parcellation analysis (videos from 40 to 60 min after drug injection) (*Fwd Loc*, forward locomotion; *Ctrl. turn*, contralateral turning). (E, M) Time course of locomotive scores. (F, N) Sum of locomotive scores in the test session. (G, O) Total dystonia scores in the same test session (sum of dystonia scores from all body regions). (H, P) Breakdown of the dystonia score into the four topographic subtypes, represented using different bar fillings (see caption). (*FL*, forelimbs; *HL*, hindlimbs, *Tr/ne*, trunk and neck). See Table S1 for statistical data. *P < 0.05, **P < 0.01, ***P < 0.001 versus AAV‐Drd2WT. [Color figure can be viewed at wileyonlinelibrary.com]

- D2R agonist treatment: Upon treatment with SUM, AAV‐Drd2KO mice did not develop any axial, limb, or orofacial AIMs (Fig. 3A; *P* < 0.01 vs. AAV‐Drd2WT at 20–100 min; *P* < 0.001 on both total score and AIM subscore analysis; Fig. 3B,C). A strong genotype effect was seen also on the locomotive scores, which were hardly detectable in the AAV‐Drd2KO animals (Fig. 3E, *P* < 0.001 for AAV‐Drd2KO vs. AAV‐Drd2WT group at 20–100 min post‐SUM injection; Fig. 3F, *P* < 0.001 on total locomotive score). The behavioral parcellation analysis revealed >90% decrease in contralateral turning in AAV‐Drd2KO mice, whereas other behaviors were not affected (Fig. 3D, *P* < 0.001 for both genotype and genotype–behavioral category interaction; *P* < 0.01 for AAV‐Drd2KO vs. AAV‐Drd2WT on ‘contralateral turn’).

SUM‐induced dystonia was totally suppressed in the AAV‐Drd2KO group (Fig. 3G,H, see also Video 1). Indeed, all dystonic features (except for tail dystonia) were significantly reduced, and tr/ne and HL scores were null in the knockout group (Fig. 3H; *P* < 0.001 for both genotype and genotype–subscore interaction; *P* < 0.01 for AAV‐Drd2KO vs. AAV‐Drd2WT on tr/ne and HL scores, *P* < 0.05 on FL scores).

- L‐DOPA treatment: Upon treatment with L‐DOPA, axial, limb, and orofacial AIMs were markedly reduced in the AAV‐Drd2KO mice compared with their controls (Fig. 3I, *P* < 0.001 for the effects of genotype and genotype–time interaction; *P* < 0.05 for AAV‐Drd2KO vs. AAV‐Drd2WT at 20–80 min post‐L‐DOPA dosing; see also Video 2). Accordingly, the total AIM score per session was reduced by approximately two‐fold in the KO group (Fig. 3J, *P* < 0.001 for AAV‐Drd2KO vs. AAV‐Drd2WT). The AIM subscore analysis revealed a highly significant difference between the two groups (Fig. 3K, *P* < 0.001 for the effects of genotype and genotype–AIM subtype interaction). Indeed AAV‐Drd2KO animals exhibited an over three‐fold reduction in axial and limb AIMs, and a milder reduction in the orofacial score (Fig. 3K, *P* < 0.001 for AAV‐Drd2KO vs. AAV‐Drd2WT on axial and limb AIMs, *P* < 0.05 on orofacial AIMs). The reduced LID severity in AAV‐Drd2KO mice concurred with larger locomotive scores in this group (Fig. 3M; *P* < 0.001 for genotype effect and genotype–time interaction; *P* < 0.05 for AAV‐Drd2KO vs. AAV‐Drd2WT at 20–60 min post‐L‐DOPA administration; Fig. 3N, *P* < 0.001 on the total locomotive score). The increase in locomotive scores reflected a reduced dyskinesia burden in the AAV‐Drd2KO group because it specifically occurred at time points when LID is most severe (cf. 20–60 min in Fig. 3I) and was accompanied by an augmented expression of normal behaviors (Fig. 3L, *P* < 0.05 for the effects of genotype and genotype–behavioral category interaction), particularly forward locomotion and rearing (*P* < 0.05 vs. AAV‐Drd2WT).

**Video 1:** Dystonic and dyskinetic features induced by a D2R agonist are suppressed by the ablation of indirect‐pathway striatal projection neuron (iSPN) D2Rs. The video shows two animals, both having a 6‐hydroxydopamine (6‐OHDA) lesion in the right hemisphere and receiving treatment with the selective D2R agonist sumanirole (SUM, 4 mg/kg, i.p.). The first clip exemplifies SUM‐induced dyskinetic‐dystonic features in a D2R‐wild‐type mouse (AAV‐D2RWT case). Note the sustained twisted postures of trunk‐neck and hindlimbs. The second clip shows an AAV‐D2RKO mouse at the same time point following drug injection (20 min, fifth day of treatment). In this case, trunk‐neck and limb dystonias are absent, and the mouse is able to move freely in the cage. Video acquired at 30 fps (1080p), real‐time playback.

**Video 2:** Dyskinetic features induced by L‐DOPA are reduced by the ablation of indirect‐pathway striatal projection neuron (iSPN) D2Rs. The video shows two animals, both having a 6‐hydroxydopamine (6‐OHDA) lesion in the right hemisphere and receiving treatment with L‐DOPA (6 mg/kg, i.p.). The first clip exemplifies L‐DOPA‐induced dyskinetic features in a D2R‐wild‐type mouse (AAV‐D2RWT case). The mouse shows hyperkinetic forelimb, orofacial and jaw movements, accompanied by axial twisting. The second clip shows an AAV‐D2RKO mouse at the same time point following drug injection (20 min, fifth day of treatment). In this case, dyskinesia is absent and the mouse moves freely in the cage. Video acquired at 30 fps (1080p), real‐time playback.

The expression of dystonic features on L‐DOPA was markedly reduced in the AAV‐Drd2KO group (Fig. 3O,P). In the dystonia subscore analysis, the KO animals showed a dramatic attenuation of HL and tr/ne components (Fig. 3P, *P* < 0.001 for genotype effect and genotype–dystonia subtype interaction; *P* < 0.05 on tr/ne and HL in the post‐hoc comparisons). In contrast, tail dystonia (which depends on D1R stimulation^23^) tended to be more prominent in the AAV‐Drd2KO group (Fig. 3P).

- D1R agonist treatment: The effects of SKF treatment in the unilateral iSPN‐D2RKO model are presented in Supplemental Results SIII and shown in Fig. S5I–P. Briefly, treatment with SKF induced the same pattern of low‐grade dyskinesia in the two genotypes, though with a total absence of axial AIMs in the AAV‐Drd2KO group. Locomotive scores and contralateral turning were not affected (Fig. S5L–N), nor was the total dystonia score (Fig. S5O). In the subscore analysis, AAV‐Drd2KO animals did not exhibit any other features than tail dystonia (Fig. S5P), thus resembling the SKF response pattern seen in Drd2KO^−/−^ mice (cf. Fig. S5H).

### 2.4 Cellular Analysis of Neuronal Activity Changes

The ribosomal protein S6 is rapidly and transiently phosphorylated in neurons following synaptic or metabolic activation.^32^ We examined the expression of Ser235/236‐phosphorylated S6 (hereinafter pS6) in BAC‐A2a‐GFP mice with 6‐OHDA lesions, receiving acute treatment with either L‐DOPA, SUM, or vehicle. The number of pS6‐positive (pS6+) cells was counted in the striatum and the external globus pallidus (GPe) on both sides of the brain. As treatment‐induced differences were minor or absent on the intact side (Fig. S6), only data from the DA‐denervated side are reported here.

**FIG. 4:**
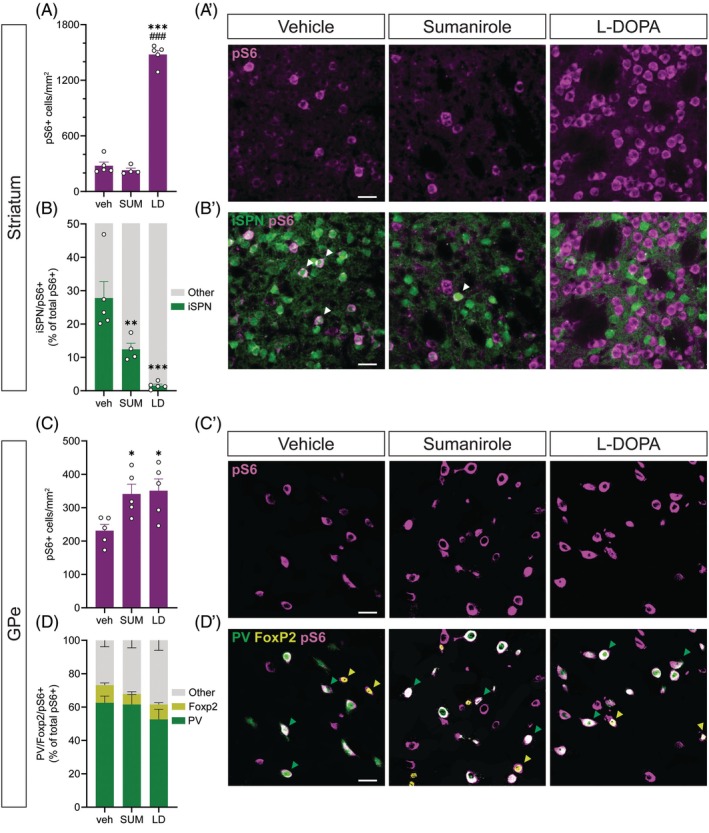
Cellular analysis of pS6 expression reveals treatment‐dependent modulations of neuronal activity in the striatum and external globus pallidus (GPe). Data are from the 6‐hydroxydopamine (6‐OHDA)‐lesioned hemisphere of A2a‐GFP (green fluorescent protein) mice acutely treated with vehicle (saline), sumanirole (SUM), or L‐DOPA (LD). (A) Number of pS6‐positive (pS6+) cells per mm^2^ in the dorsolateral striatum. (A') The corresponding representative photomicrographs. (B) Percentage of indirect‐pathway striatal projection neurons (iSPNs) (GFP‐labeled, green bars) among the total number of pS6^+^ cells. (B') Representative photomicrographs, where arrowheads indicate pS6/GFP double‐labeled cells (scale bar: 200 μm). (C) Number of pS6+ cells per mm^2^ in the dorsolateral GPe. (C') Representative photomicrographs. (D) Percentage of pS6+ cells colocalized with parvalbumin (PV, green) or the transcription factor FoxP2 (yellow). (D') Representative photomicrographs; green and yellow arrowheads indicate pS6+ cells double‐labeled for PV or FoxP2, respectively. See Table S1 for ANOVA data. *P < 0.05, **P < 0.01, ***P < 0.001 versus vehicle; ^###^ *P* < 0.001 versus SUM. [Color figure can be viewed at wileyonlinelibrary.com]

In the dorsal striatum, L‐DOPA induced approximately a six‐fold increase in the number of pS6+ neurons, whereas SUM did not differ from vehicle (Fig. 4A,A'; *P* < 0.001 for L‐DOPA vs. both vehicle and SUM). Although ≥20% of pS6+ neurons were positive for GFP after vehicle injection, only 12% and 1.5% showed GFP colocalization upon treatment with SUM and L‐DOPA, respectively (Fig. 4B,B'; *P* < 0.01 vs. vehicle in both drug‐treated groups). These data show that the vast majority of striatal neurons activated by SUM and L‐DOPA are not iSPNs. We also examined the expression of pS6 in cholinergic interneurons (identified using the ChAT antibody) but did not find any significant treatment effect in this cell population (data not shown). We therefore conclude that both L‐DOPA and SUM had activated pS6 predominantly in dSPNs, though with a dramatic quantitative difference between the two dopaminergic agents. Furthermore, as both L‐DOPA and SUM significantly reduced the extent of pS6‐GFP colocalization, we conclude that both treatments had inhibited the activity of iSPNs (in keeping with iSPN calcium imaging data from this mouse model^33^).

We hypothesized that reduced iSPN activity would disinhibit prototypical GPe neurons, which are a prominent target of iSPN GABAergic projections and make up nearly 70–75% of all GPe cells.^34^ Consistent with our hypothesis, the number of pS6+ neurons in the DA‐denervated GPe was significantly increased after the administration of L‐DOPA or SUM (Fig. 4C; *P* < 0.05 vs. vehicle for both treatments). To explore the cell type‐specificity of this effect, we examined the colocalization of pS6 with markers of prototypical and arkypallidal GPe neurons, that is, the calcium‐binding protein parvalbumin (PV) and the transcription factor forkhead box protein P2 (FoxP2), respectively. The extent of pS6 colocalization with PV or FoxP2 was not significantly altered by the treatments: the majority (>50%) of pS6+ neurons expressed PV, whereas the colocalization with FoxP2 was modest (6–10%) (Fig. 4D,D'; *P* < 0.001 for cell type marker, *P* > 0.05 for both treatment and treatment–cell type interaction). Taken together, these results show that the administration of L‐DOPA and SUM is followed by neuronal activation in the DA‐denervated GPe, with a prominent recruitment of PV‐positive prototypical neurons.

### 2.5 Ablation of iSPN‐D2Rs Prevents Pallidal Activity Changes Induced by L‐DOPA and SUM

**FIG. 5:**
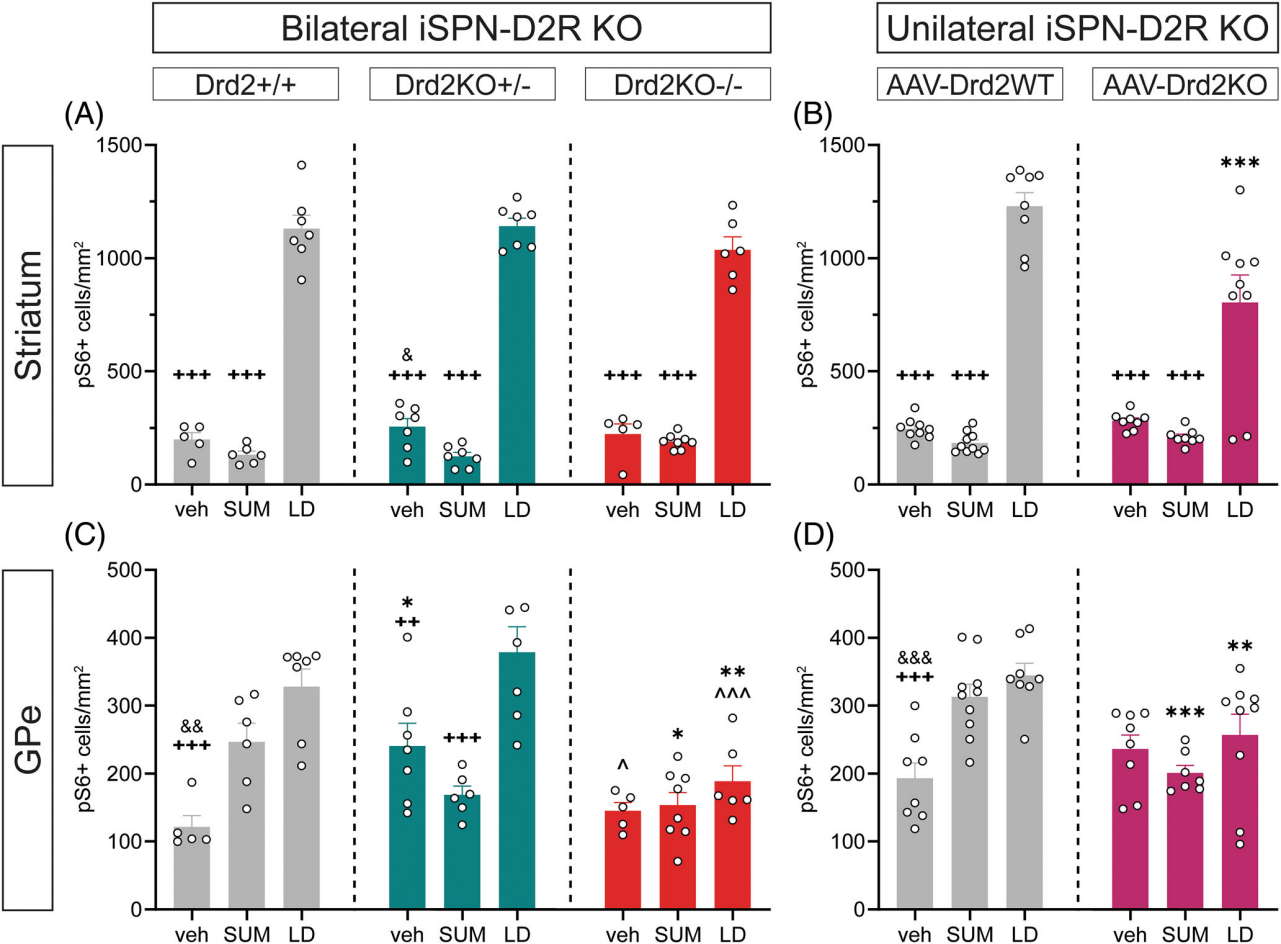
Impact of indirect‐pathway striatal projection neuron (iSPN) D2R deletion on treatment‐induced pS6 activation in the striatum (upper row) and external globus pallidus (GPe) (lower row). Data are from the dopamine (DA)‐denervated hemisphere of animals treated with vehicle (veh), sumanirole (SUM), or L‐DOPA (LD). (A, B) Striatal numbers of pS6+ cells per mm^2^ in the bilateral and unilateral iSPN‐D2R knockout (KO) model, respectively, along with the corresponding control groups. (C, D) Pallidal number of pS6+ cells per mm^2^ in the bilateral and unilateral iSPN‐D2R KO model and the corresponding controls. See Table S1 for ANOVA data. *P < 0.05, **P < 0.01, ***P < 0.001 versus the corresponding D2R‐wild‐type control for the same treatment (ie, Drd2^+/+^ in A, C or AAV‐Drd2WT in B, D); ^^^*P* < 0.001 for Drd2^−/−^ versus Drd2^+/−^ for same treatment; ^+^ *P* < 0.05, ^++^ *P* < 0.01, ^+++^ *P* < 0.001 versus LD for same genotype; ^&^ *P* < 0.05, ^&&&^ *P* < 0.001 versus SUM for the same genotype. [Color figure can be viewed at wileyonlinelibrary.com]

We next examined the impact of iSPN‐D2R ablation on striatal and pallidal pS6 expression. To this end, we used striatal and pallidal sections from the mice shown in Figs. 2 and 3, complemented with new animals prepared specifically for this analysis. As shown in Fig. 5A, treatment with L‐DOPA but not SUM markedly raised the number of pS6+ neurons in the DA‐denervated striatum, and this response was not significantly affected in the bilateral iSPN‐D2R KO model (see Drd2KO^−/−^ in Fig. 5A; *P* < 0.001 for treatment effect, *P* > 0.05 for both genotype and treatment–genotype interaction; *P* < 0.001 for L‐DOPA vs. both vehicle and SUM within each genotype). However, AAV‐Drd2KO mice showed an approximately 35% reduction in the striatal number of pS6+ neurons on L‐DOPA (Fig. 5B; *P* < 0.001 for treatment effect, *P* < 0.05 for genotype effect, *P* < 0.001 for treatment–genotype interaction; *P* < 0.001 for AAV‐Drd2KO vs. AAV‐Drd2WT on L‐DOPA).

We also counted the number of pS6‐positive neurons in the DA‐denervated GPe, In Drd2^+/+^ mice, pallidal pS6+ cell numbers were significantly increased following L‐DOPA and SUM administration (see grey bars in Fig. 5C). This response was strongly impacted by the iSPN‐D2R ablation (Fig. 5C; *P* < 0.001 for genotype effect, *P* < 0.01 for treatment–genotype interaction). Hemizygous Drd2KO^+/−^ mice showed an increased pS6+ expression after L‐DOPA though not SUM treatment (Fig. 5C, mid panel, *P* < 0.001 for SUM vs. L‐DOPA, within‐genotype comparison). Homozygous Drd2KO^−/−^ animals did not show any significant increase in pallidal pS6 cell numbers in response to either SUM or L‐DOPA (Fig. 5C; right panel, *P* < 0.01 for Drd2KO^−/−^ vs. both other groups on L‐DOPA; *P* < 0.05 vs. Drd2^+/+^ on SUM). Likewise, AAV‐Drd2KO mice did not show any significant increase in pallidal pS6+ cell numbers following either L‐DOPA or SUM treatment (Fig. 5D; *P* < 0.01 for the effects of treatment, genotype, treatment–genotype interaction; *P* < 0.01 for AAV‐Drd2KO vs. AAV‐Drd2WT on both SUM and L‐DOPA).

## 3 Discussion

The expression of LID is associated with a large increase in the activity of dSPNs and a concomitant decrease in the activity of iSPNs.^7^, ^33^, ^35^, ^36^ These effects are attributed to the facilitatory and inhibitory action of DA receptor stimulation on dSPNs and iSPNs, respectively. Chemogenetic and optogenetic studies in rodent models have established that the abovementioned changes in SPN activity are causal to the appearance of involuntary movements (reviewed in Cenci and Kumar^37^). Accordingly, cell type‐specific iSPN stimulation improves LID.^22^, ^38^, ^39^ It is therefore conceivable that D2R antagonists exert antidyskinetic effects by lifting the inhibitory tone of DA on iSPNs. However, D2Rs are expressed in several cell types beyond iSPNs, including dopamine neurons, cortical neurons, and striatal interneurons, highlighting the need for specific investigations of D2R signaling in iSPNs.

Our results provide the first demonstration that iSPN‐D2Rs in the dorsal striatum significantly contribute to LID, and that they totally account for the dyskinetic and dystonic responses evoked by pharmacological D2R stimulation (mainly, twisting movements and abnormal postures of trunk, neck, and limbs). In contrast, iSPN‐D2Rs do not appear to be necessary for the expression of D1 agonist‐induced dyskinesia or dystonia (mainly tail dorsiflexion), nor do they contribute to D1 agonist‐induced turning. The clearcut reduction in L‐DOPA‐induced AIMs observed in both of our iSPN‐D2R knockout models is at variance with previous reports showing that ablating D2Rs, whether globally^6^, ^8^ or specifically in iSPNs,^9^ has either no effect or an aggravating effect on L‐DOPA‐induced axial, limb, and orofacial AIMs (the rodent equivalent of LID). These conflicting results most likely depend on the use of different D2R KO models. Previous studies have used animal models where the ablation of D2R signaling started during embryonic development (see Supplemental Discussion SII). In contrast, our unilateral iSPN‐D2R KO model lost iSPN‐D2Rs at a fully mature adult age, and our bilateral KO model most likely lost iSPN‐D2Rs during the first postnatal weeks. Indeed, the adenosine A2a gene starts to be expressed in rodent SPNs during the early postnatal period, and its expression levels increase from the second week of life concomitantly with the acquisition of a mature SPN phenotype.^40^ It is important to reflect on these temporal aspects because an early ablation of important genes can lead to developmental compensations masking the functional consequences of the gene defect.^41^ In particular, a study comparing mice with constitutive or inducible D2R gene deletion reported that compensatory mechanisms occurred only in the constitutive model, being absent in animals rendered D2R‐deficient as adults.^42^ Of relevance to our study, D2R deletion in iSPNs has been reported to cause a compensatory augmentation of D1R signaling.^43^ Such a phenomenon might underlie the increased striatal expression of D1R signaling markers (c‐Fos, phosphorylated ERK) detected in the iSPN‐D2R KO model that also showed increased LID severity in a previous study.^9^ These D1 signaling markers are upregulated in dSPNs and causally contribute to the development of LID (recently reviewed in Cenci and Ferrario^44^). Compensatory adaptations to the loss of iSPN‐D2R signaling most likely occurred also in our KO models.^20^, ^43^ However, the striatal upregulation of pS6 induced by L‐DOPA (also a marker of supersensitive D1 signaling^45^) was not augmented but rather decreased in our iSPN‐D2R KO models. In addition, upon ablating iSPN‐D2Rs using two different approaches we obtained strikingly similar behavioral–pharmacological outcomes, in particular: (i) the severity of L‐DOPA‐induced AIMs and tr/ne dystonia was halved, whereas locomotive scores and non‐pathological behaviors were either unchanged or improved and (ii) dyskinetic, dystonic, and turning responses associated with selective D2R stimulation were completely abolished, whereas those induced by a D1‐class agonist remained unaffected. The consistency of these results across two very different KO models makes us confident that they indeed stem from the loss of D2R function in iSPNs.

As a marker of treatment‐dependent changes in neuronal activity, we used ribosomal protein S6, a component of the 40S ribosomal subunit in all eukaryotic cells undergoing rapid phosphorylation in response to increased metabolic demands.^32^ Using pS6 as a marker allows for detecting neuronal activity changes induced by both L‐DOPA and D2‐class agonists.^31^, ^32^ In D2R‐wild‐type mice with 6‐OHDA lesions, L‐DOPA largely increased pS6 expression in the DA‐denervated striatum and produced a significant pS6 upregulation also in the GPe. In contrast, SUM raised pS6 levels only in the GPe. After both treatments, over 50% of the pS6+ cells in the GPe were prototypical PV neurons, a cell population receiving strong inhibitory inputs from iSPNs.^46^ As iSPN activity was decreased by both L‐DOPA and SUM (cf. pS6‐GFP colocalization data), we hypothesize that the upregulation of pS6 in the GPe reflected the lifting of a D2R‐dependent, striatally derived GABAergic inhibition. In agreement with this hypothesis, the upregulation of pallidal pS6+ cell numbers by both L‐DOPA and SUM was non‐significant in the iSPN‐D2R KO models. Importantly, a recent study has reported that prototypical PV‐positive neurons in the GPe show increased activity in LID and causally contribute to the expression of dyskinesia (mainly the axial component).^47^ It is therefore likely that iSPN‐D2R signaling mediates D2‐dependent dyskinetic and dystonic features via disinhibition of prototypical GPe neurons. In addition, the blunted striatal upregulation of pS6 on L‐DOPA in the viral KO model suggests that the decreased LID severity also depends on stronger collateral inhibition of dSPNs by iSPNs resulting from the loss of D2R signaling. Relevant to this proposition is the finding that iSPN‐D2R ablation causes an increased synaptic GABAergic transmission onto dSPNs.^20^ The physiological collateral inhibition exerted by iSPNs on dSPNs is ‐ likely reduced after the administration of L‐DOPA, contributing to dSPN hyperactivity in LID.^35^, ^36^

In conclusion, our results establish that the D2R pool expressed in iSPNs causally contributes to LID and mediates D2R‐dependent dystonic features. Dystonia is part of the spectrum of treatment‐dependent dyskinesias in Parkinson's disease^15^, ^48^ and contributes to reversible postural abnormalities that are reportedly more frequent in patients receiving D2R‐class agonist treatment.^49^, ^50^, ^51^ By revealing the critical role of iSPN D2Rs, our results pave the way for an exploration of downstream molecular and circuit mechanisms informing the development of new therapeutic targets.

## Author Roles

(1) Research Project: A. Conception, B. Organization, C. Execution; (2) Statistical Analysis: A. Design, B. Execution, C. Review and Critique; (3) Manuscript: A. Writing of the First Draft, B. Review and Critique.

L.A.: 1A, 1B, 1C, 2A, 2B, 2C, 3A.

T.N.: 1B, 1C, 2A, 2B, 2C, 3B.

E.E.: 1B, 1C, 2B, 2C, 3B.

J.J.: 1C, 3B.

O.F.E.: 1C, 2B, 2C, 3B.

M.A.C.: 1A, 1B, 2A, 2C, 3A, 3B.

## Financial Disclosures of All Authors (for the Past 12 Months)

Stock Ownership in medically related fields, Intellectual Property Rights, Consultancies, Expert Testimony, Partnerships, Honoraria, Royalties, Other: None. Advisory Boards: Swedish Brain Foundation (M.A.C., J.J.). Employment: Lund University (T.N., E.E., O.F.E., J.J., M.A.C.). Research Grants: Swedish Research Council (M.A.C., J.J.); Swedish Brain Foundation (M.A.C., J.J.); Swedish Government Funding for Clinical Research (M.A.C.); Swedish Parkinson Foundation (M.A.C.); Swedish Cancer Foundation (J.J.); Trolle‐Wachtmeister Foundation (J.J.); ASAP/The Michael J. Fox Foundation (J.J., M.A.C.); CCXDP (J.J.); Lundbeck Foundation (M.A.C., by way of Harwig Siebner); National Institutes of Health (NIH)/National Institute of Neurological Disorders and Stroke (NINDS) (M.A.C., by way of David Eidelberg and Corey Hopkins); The Royal Physiographic Society of Lund (O.F.E.); Anna‐Lisa Rosenberg Foundation (O.F.E.).

## Supporting information


**Data S1.** Supporting Information.

**Figure S1.** Motor phenotype in mice with bilateral iSPN‐D2R ablation before or after a 6‐OHDA lesion. Open‐field motions over 120 min and cylinder test were recorded under drug‐free conditions both before (A–F') and after (G–L') a 6‐OHDA lesion in the right MFB. Data show comparisons between mice with normal levels of D2R in iSPNs (Drd2^+/+^, n = 13) and hemizygous (Drd2KO^+/−^, n = 14) or homozygous iSPN‐D2R knockout mice (Drd2KO^−/−^, n = 13/12). (A, G) Percentage of right turns (ipsilateral to the side to be lesioned). (B, H) Cylinder test, percentage of contralateral forelimb use. (C, I) Sum of immobility episodes (≥3 s duration/episode) in the recording session. (D, J) Time course of rearing events and (D', J') sum of rearing events. (E, K) Time course of distance travelled in open‐field test and (E', K') sum of distance travelled during the test. (F, L) Time course of motion speed (max speed/bin) and (F', L') average max‐speed values in the recording session. (M) Motion traces during a 2‐hr exploration of the open field arena before and after the 6‐OHDA lesion. Yellow squares demarcate the center of the arena versus the periphery. (N) Ratio of time spent in the center versus the periphery (grey shade shows DA‐intact condition). Data are represented as mean ± SEM (line diagrams) or box and whiskers. Kruskal–Wallis test with Dunn's post‐hoc comparisons. *P < 0.05, **P < 0.01, ***P < 0.001 (vs. Drd2^+/+^). ^*P* < 0.05, ^^*P* < 0.01 (Drd2^+/−^ vs. Drd2^−/−^) or Mann–Whitney U test. ^&^ *P* < 0.05 (vs. DA‐intact).
**Figure S2.** Motor phenotype in mice with unilateral iSPN‐D2R ablation before or after a 6‐OHDA lesion. Open‐field motions over 120 min and cylinder test were recorded under drug‐free conditions both before (A–F') and after (G–L') a 6‐OHDA lesion in the right MFB. Data show comparisons between Drd2^loxP‐/loxP‐^ (AAV‐Drd2WT; n = 8) or Drd2^loxP+/loxP+^ (AAV‐Drd2KO; n = 12) mice injected with the AAV‐PENK‐Cre construct in the right dorsolateral striatum. (A, G) Percentage of right turns (ipsilateral to the side to be lesioned). (B, H) Cylinder test‐percentage of left forelimb use. (C, I) Sum of immobility episodes (≥3 s duration/episode) in the recording session. (D, J) Time course of rearing events and (D', J') sum of rearing events. (E, K) Time course of distance travelled in the open‐field test and (E', K') sum of distance travelled during the test. (F, L) Time course of motion speed, expressed as max speed/recording bin and (F', L') average max‐speed values in the recording session. (M) Motion traces during a 2‐hr exploration of the open field arena both before and after a 6‐OHDA lesion. Yellow squares demarcate the center of the arena versus the periphery. The ratio of time spent in the center versus the periphery of the arena is reported in panel (N) where the DA‐intact condition is indicated with the grey shaded area. Data are represented as mean ± SEM (line diagrams) or box and whiskers. Mann–Whitney U test. *P < 0.05, **P < 0.01, ***P < 0.001 (WT vs. KO). ^&&^ *P* < 0.01 (vs. DA‐intact).
**Figure S3.** Evolution of dyskinesia scores over the drug treatments in mice with bilateral ablation of iSPN D2Rs. Animals correspond to Figure 2 in the main article. Data are reported over days 1, 3, and 5 of treatment with the D2R‐class agonist sumanirole (4 mg/kg) (A–D), the D1‐class agonist SKF38393 (3.0 mg/kg) (E–H) and L‐DOPA (6.0 mg/kg) (I–L). (A) Sum of abnormal involuntary movement (AIM) scores per session over days 1, 3, and 5 of sumanirole treatment. Two‐way repeated measures (RM) ANOVA; Genotype: *F*_(2, 20)_ 72.95, *P* < 0.001; Day: *F*_(1.391, 27.82)_ 7.49, *P* = 0.0058; Interaction: *F*_(2.782, 27.82)_ 2.23, *P* = 0.1108. Tukey's post‐hoc comparisons: ***P < 0.001 versus Drd2^−/−^, ^#^ *P* < 0.05, ^##^ *P* < 0.01, and ^###^ *P* < 0.001 versus Drd2^+/−^, ^*P* < 0.05 Drd2^+/−^ versus Drd2^−/−^. (B) Time course of axial, limb, and orofacial AIM scores (sum per monitoring period) on day 1 after sumanirole injection. Two‐way RM ANOVA; Genotype: *F*_(2, 20)_ 82.22, *p* < 0.0001; Day: *F*_(3.815, 76.30)_ 24.04, *P* < 0.0001; Interaction: *F*_(7.630, 76.30)_ 18.01, *P* < 0.0001. Tukey's post‐hoc comparisons: **P < 0.01 and ***P < 0.001 versus Drd2^−/−^, ^##^ *P* < 0.01 and ^###^ *P* < 0.001 versus Drd2^+/−^. (C) Time course of axial, limb, and orofacial AIM scores (sum per monitoring period) on day 3 after sumanirole injection. Two‐way RM ANOVA; Genotype: *F*_(2, 20)_ 20.25, *P* < 0.0001; Day: *F*_(4.102, 82.03)_ 8.234, *P* < 0.0001; Interaction: *F*_(8.203, 82.03)_ 3.144, *P* = 0.0035. Tukey's post‐hoc comparisons: *P < 0.05, **P < 0.01, and ***P < 0.001 versus Drd2^−/−^, ^##^ *P* < 0.01 versus Drd2^+/−^. (D) Time course of axial, limb, and orofacial AIM scores (sum per monitoring period) on day 5 after sumanirole injection. Two‐way RM ANOVA; Genotype: *F*_(2, 20)_ 36.96, *P* < 0.0001; Day: *F*_(2.392, 47.85)_ 27.09, *P* < 0.0001; Interaction: *F*_(4.785, 47.85)_ 10.49, *P* < 0.0001. Tukey's post‐hoc comparisons: *P < 0.05 and **P < 0.001 versus Drd2^−/−^, ^#^ *P* < 0.05, ^##^ *P* < 0.01, and ^###^ *P* < 0.001 versus Drd2^+/^. (E) Sum of AIM scores per session over day 1, 3 and 5 of SKF treatment. Two‐way RM ANOVA; Genotype: *F*_(2, 20)_ 2.750, *P* = 0.001; Day: *F*_(1.530, 30.60)_ 3.542, *P* = 0.0525; Interaction: *F*_(3.060, 30.60)_ 1.462, *P* = 0.2440. (F) Time course of axial, limb, and orofacial AIM scores (sum per monitoring period) on day 1 after SKF injection. Two‐way RM ANOVA; Genotype: *F*_(2, 20)_ 2.933, *P* = 0.0764; Day: *F*_(2.167, 43.34)_ 8. 684, *P* = 0.0006; Interaction: *F*_(4.334, 43.34)_ 0.9642, *P* = 0.4417. (G) Time course of axial, limb, and orofacial AIM scores (sum per monitoring period) on day 3 after SKF injection. Two‐way RM ANOVA; Genotype: *F*_(2, 20)_ 3.001, *P* = 0.0725; Day: *F*_(1.839, 36.78)_ 15.69, *P* < 0.0001; Interaction: *F*_(3.678, 36.78)_ 1.309, *P* = 0.2854. (H) Time course of axial, limb, and orofacial AIM scores (sum per monitoring period) on day 5 after SKF injection. Two‐way RM ANOVA; Genotype: *F*_(2, 20)_ 0.3268, *P* = 0.7250; Day: *F*_(2.391, 47.47)_ 19.24, *P* < 0.0001; Interaction: *F*_(14, 139)_ 0.7019, *P* = 0.7696. (I) Sum of AIM scores per session over days 1, 3, and 5 of L‐DOPA treatment. Two‐way RM ANOVA; Genotype: *F*_(2, 20)_ 7.683, *P* = 0.0030; Day: *F*_(1.599, 31.98)_ 4.386, *P* = 0.0278; Interaction: *F*_(3.198, 31.98)_ 1.687, *P* = 0.1871. Tukey's post‐hoc comparisons: **P < 0.01 versus Drd2^−/−^; ^&^ *P* < 0.05 Drd2^−/−^ versus day 1. (J) Time course of axial, limb, and orofacial AIM scores (sum per monitoring period) on day 1 after L‐DOPA injection. Two‐way RM ANOVA; Genotype: *F*_(2, 20)_ 6.848, *P* = 0.0054; Day: *F*_(2.570, 51.40)_ 58.43, *P* < 0.0001; Interaction: *F*_(5.140, 51.40)_ 3.474, *P* = 0.0083. Tukey's post‐hoc comparisons: *P < 0.05 and **P < 0.01 versus Drd2^−/−^. (K) Time course of axial, limb, and orofacial AIM scores (sum per monitoring period) on day 3 after L‐DOPA injection. Two‐way RM ANOVA; Genotype: *F*_(2, 20)_ 3.078, *P* = 0.0683; Day: *F*_(3.557, 71.14)_ 74.26, *P* < 0.0001; Interaction: *F*_(7.114, 71.14)_ 1.297, *P* = 0.2640. (L) Time course of axial, limb, and orofacial AIM scores (sum per monitoring period) on day 5 after L‐DOPA injection. Two‐way RM ANOVA; Genotype: *F*_(2, 20)_ 4.189, *P* = 0.0302; Day: *F*_(2.023, 40.47)_ 63.13, *P* < 0.0001; Interaction: *F*_(12, 120)_ 1.626, *P* = 0.0930.
**Figure S4.** Evolution of dyskinesia scores over the drug treatments in mice with unilateral ablation of iSPN D2Rs. Animals correspond to Figure 3 in the main article. Data are reported over days 1, 3, and 5 of treatment with the D2R‐class agonist sumanirole (4 mg/kg) (A–D), the D1‐class agonist SKF38393 (3.0 mg/kg) (E–H), and L‐DOPA (6.0 mg/kg) (I–L). (A) Sum of abnormal involuntary movement (AIM) scores per session over days 1, 3, and 5 of sumanirole treatment. Two‐way repeated measures (RM) ANOVA; Genotype: *F*_(1, 19)_ 170.4, *P* < 0.0001; Day: *F*_(1.783, 33.88)_ 31.64, *P* < 0.0001; Interaction: *F*_(1.783, 33.88)_ 31.64, *P* < 0.0001. Tukey's post‐hoc comparisons: ***P < 0.001 versus Drd2^+/+^, ^&&&^ *P* < 0.001, Drd2^+/+^ versus day 1 of the same genotype. (B) Time course of axial, limb, and orofacial AIM scores (sum per monitoring period) on day 1 after sumanirole injection. Two‐way RM ANOVA; Genotype: *F*_(1, 19)_ 58.79, *P* < 0.0001; Day: *F*_(3.360, 63.84)_ 19.17, *P* < 0.0001; Interaction: *F*_(3.360, 63.84)_ 19.17, *P* < 0.0001. Tukey's post‐hoc comparisons: *P < 0.05, **P < 0.01, and ***P < 0.001 versus Drd2^−/−^. (C) Time course of axial, limb, and orofacial AIM scores (sum per monitoring period) on day 3 after sumanirole injection. Two‐way RM ANOVA; Genotype: *F*_(1, 19)_ 172.0, *P* < 0.0001; Day: *F*_(3.970, 75.42)_ 28.27, *P* < 0.0001; Interaction *F*_(3.970, 75.42)_ 28.27, *P* < 0.0001. Tukey's post‐hoc comparisons: *P < 0.05 and **P < 0.001 versus Drd2^−/−^. (D) Time course of axial, limb, and orofacial AIM scores (sum per monitoring period) on day 5 after sumanirole injection. Two‐way RM ANOVA; Genotype: *F*_(1, 19)_ 100.7, *P* < 0.0001; Day: *F*_(2.697, 51.24)_ 30.70, *P* < 0.0001; *F*_(2.697, 51.24)_ 30.70, *P* < 0.0001. Tukey's post‐hoc comparisons: **P < 0.01 and ***P < 0.001 versus Drd2^−/−^. (E) Sum of AIM scores per session over days 1, 3, and 5 of SKF treatment. Two‐way RM ANOVA; Genotype: *F*_(1, 19)_ 23.58, *P* = 0.0001; Day: *F*_(1.494, 28.38)_ 7.547, *P* = 0.0048; Interaction: *F*_(1.494, 28.38)_ 12.96, *P* = 0.0003. ***P < 0.001 versus Drd2^−/−^, ^&&^ *P* < 0.01, Drd2^+/+^ versus day 1 of the same genotype. (F) Time course of axial, limb, and orofacial AIM scores (sum per monitoring period) on day 1 after SKF injection. Two‐way RM ANOVA; Genotype: *F*_(1, 19)_ 33.57, *P* < 0.0001; Day: *F*_(2.390, 45.41)_ 48.89, *P* < 0.0001; Interaction: *F*_(2.390, 45.41)_ 16. 37, *P* < 0.0001. *P < 0.05 versus Drd2^−/−^. (G) Time course of axial, limb, and orofacial AIM scores (sum per monitoring period) on day 3 after SKF injection. Two‐way RM ANOVA; Genotype: *F*_(1, 19)_ 2.294, *P* = 0.1463; Day: *F*_(1.885, 35.82)_ 29.13, *P* < 0.0001; Interaction: *F*_(1.885, 35.82)_ 2.137, *P* = 0.1353. (H) Time course of axial, limb, and orofacial AIM scores (sum per monitoring period) on day 5 after SKF injection. Two‐way RM ANOVA; Genotype: *F*_(1, 19)_ 4.114, *P* = 0.0568; Day: *F*_(1.994, 37.88)_ 42.62, *P* < 0.0001; Interaction: *F*_(1.994, 37.88)_ 2.217, *P* = 0.1230. (I) Sum of AIM scores per session over days 1, 3, and 5 of L‐DOPA treatment. Two‐way RM ANOVA; Genotype: *F*_(1, 19)_ 60.15, *P* < 0.0001; Day: *F*_(1.426, 27.10)_ 3.439, *P* = 0.0609; Interaction: *F*_(1.426, 27.10)_ 0.900, *P* = 0.3866. Tukey's post‐hoc comparisons: ***P < 0.01 versus Drd2^−/−^. (J) Time course of axial, limb, and orofacial AIM scores (sum per monitoring period) on day 1 after L‐DOPA injection. Two‐way RM ANOVA; Genotype: *F*_(1, 19)_ 20.91, *P* = 0.0002; Day: *F*_(2.632, 50.00)_ 63.52, *P* < 0.0001; Interaction: *F*_(2.632, 50.00)_ 7.319, *P* = 0.0006. Tukey's post‐hoc comparisons: *P < 0.05 and **P < 0.01 versus Drd2^−/−^. (K) Time course of axial, limb, and orofacial AIM scores (sum per monitoring period) on day 3 after L‐DOPA injection. Two‐way RM ANOVA; Genotype: *F*_(1, 19)_ 42.69, *P* < 0.0001; Day: *F*_(2.318, 44.05)_ 77.00, *P* < 0.0001; Interaction: *F*_(2.318, 44.05)_ 19.56, *P* < 0.0001. *P < 0.05, **P < 0.01, and ***P < 0.001 versus Drd2^−/−^. (L) Time course of axial, limb, and orofacial AIM scores (sum per monitoring period) on day 5 after L‐DOPA injection. Two‐way RM ANOVA; Genotype: *F*_(1, 19)_ 43.95, *P* < 0.0001; Day: *F*_(2.224, 42.26)_ 136.1, *P* < 0.0001; Interaction: *F*_(2.224, 42.26)_ 13.81, *P* < 0.0001. *P < 0.05, **P < 0.01, and ***P < 0.001 versus Drd2^−/−^.
**Figure S5.** Dyskinetic and dystonic features induced by a D1 receptor agonist are not significantly attenuated by the ablation of iSPN D2Rs. Data are from the last treatment day with the D1R‐class agonist SKF38393 (3.0 mg/kg) for the bilateral (A–H) and unilateral (I–P) iSPN‐D2R knockout models. (A) Time course of axial, limb, and orofacial AIM scores (sum per monitoring period). Two‐way repeated measures (RM) ANOVA; Genotype: *F*_(2, 20)_ 0.33, *P* = 0.33; Interaction: *F*_(14, 139)_ 0.70, *P* = 0.7019. (B) Sum of axial, limb, and orofacial AIM scores in the corresponding test session. Kruskal–Wallis test; KW(genotype) = 0.18, *P* = 0.9122. (C) Breakdown of the abnormal involuntary movement (AIM) scores into the three dyskinesia subtypes, represented using different bar fillings (see caption). Two‐way RM ANOVA; Genotype: *F*_(2, 20)_ 0.28, *P* = 0.7612; Interaction: *F*_(2.6, 26.0)_ 0.50, *P* = 0.6588. (D) Other behavior (contralateral turning, forward locomotion, grooming, and rearing) at the peak of dyskinesia (40 and 60 min after drug injection) as measured with the event‐recording software JWatcher. Two‐way RM ANOVA; Genotype: *F*_(2, 20)_ 4.56, *P* < 0.05; Interaction: *F*_(2.9, 29.2)_ 4.10, *P* < 0.05. Tukey's post‐hoc comparisons: *P < 0.05 (vs. −/−). (E) Locomotive scores recorded during the corresponding test sessions. Two‐way RM ANOVA; Genotype: *F*_(2, 20)_ 5.94, *P* < 0.01; Interaction: *F*_(8.5, 84.0)_ 1.81, *P* = 0.0823. (F) Sum of locomotor scores in the corresponding test session. Kruskal–Wallis test; KW(genotype) = 10.03, *P* < 0.01. Dunn's post‐hoc comparisons: *P < 0.05 (vs. Drd2^+/+^), ^*P* < 0.05 (Drd2^+/−^ vs. Drd2^−/−^). (G) Total dystonia scores in the corresponding test session (sum of dystonia scores for trunk/neck, contralateral hindlimb, ipsilateral hindlimb, contralateral forelimb, ipsilateral forelimb, and tail). Kruskal–Wallis test; KW (genotype) = 4.54, *P* = 0.1031. (H) Breakdown of the dystonia scores into the four topographic subtypes, represented using different bar fillings (see caption). For this representation, the scores recorded from hindlimbs and forelimbs on both sides were summed and named HL and FL, respectively. Two‐way RM ANOVA; Genotype: *F*_(2, 20)_ 2.73, *P* = 0.0893; Interaction: *F*_(6, 60)_ 4.08, *P* < 0.01. (I) Time course of axial, limb, and orofacial AIM scores (sum per monitoring period). Two‐way RM ANOVA; Genotype: *F*_(1, 19)_ 4.11, *P* = 0.0568; Interaction: *F*_(8, 152)_ 2.22, *P* < 0.05. (J) Sum of axial, limb, and orofacial AIM scores in the corresponding test session. Mann–Whitney U test; *P* = 0.1061. (K) Breakdown of the AIM scores into the three dyskinesia subtypes, represented using different bar fillings (see caption). Two‐way RM ANOVA; Genotype: *F*_(1, 19)_ 4.11, *P* = 0.0568; Interaction: *F*_(2, 38)_ 1.49, *P* = 0.2382. (L) Other behavior (contralateral turning, forward locomotion, grooming, and rearing) at the peak of dyskinesia (40 and 60 min after drug injection) as measured with the event‐recording software JWatcher. Two‐way RM ANOVA; Genotype: *F*_(1, 18)_ 0.027, *P* = 0.8720; Interaction: *F*_(1.8, 31.9)_ 1.28, *P* = 0.2891. (M) Locomotive scores recorded during the corresponding test sessions. Two‐way RM ANOVA; Genotype: *F*_(1, 19)_ 0.95, *P* = 0.3423; Interaction: *F*_(6, 114)_ 1.01, *P* = 0.4242. (N) Sum of locomotor scores in the corresponding test session. Mann–Whitney U test; *P* = 0.4037. (O) Total dystonia scores in the corresponding test session (sum of dystonia scores for trunk/neck, contralateral hindlimb, ipsilateral hindlimb, contralateral forelimb, ipsilateral forelimb, and tail). Mann–Whitney U test; *P* = 0.6343. (P) Breakdown of the dystonia scores into the four topographic subtypes, represented using different bar fillings (see caption). For this representation, the scores recorded from hindlimbs and forelimbs on both sides were summed and named HL and FL, respectively. Two‐way RM ANOVA; Genotype: *F*_(1, 19)_ 0.82, *P* = 0.3778; Interaction: *F*_(3, 57)_ 0.30, *P* = 0.8266. Data are represented as mean ± SEM or box and whiskers.
**Figure S6.** Comparison of pS6‐positive cell numbers between intact and DA‐denervated sides. These data were obtained from the unilaterally 6‐OHDA‐lesioned animals with intact D2R signaling represented in Fig. 4. The results show that treatment with L‐DOPA or the D2R agonist sumanirole significantly increased the number of pS6‐positive neurons in the dorsal striatum and the external globus pallidus (GPe) on the DA‐denervated (purple bars) but not the intact hemisphere (pink bars). Veh, vehicle (saline); SUM, sumanirole; LD, L‐DOPA. (A) Two‐way ANOVA: Lesion: *F*_(1, 11)_ 93.70, *P* < 0.001; Treatment: *F*_(2, 11)_ 64.69, *P* < 0.001; Interaction: *F*_(2, 11)_ 137.6, *P* < 0.0001. (B) Two‐way ANOVA: Lesion: *F*_(1, 12)_ 2.01, *P* = 0.1815; Treatment: *F*_(2, 12)_ 1.13, *P* = 0.3561; Interaction: *F*_(2, 12)_ 5.08, *P* < 0.05. Data are represented as mean ± SEM. Tukey's post‐hoc comparisons. *P < 0.05, ***P < 0.001 (intact vs. lesion for same treatment) ^*##*^ *P* < 0.01, ^###^ *P* < 0.001 (vs. veh for same hemisphere) ^^^*P* < 0.001 (vs. SUM for same hemisphere).
**Table S1.** Detailed statistical information for data presented in Figs. 2, 3, 1, 2, 4, 5. RM, repeated measures.

## Data Availability

The data that support the findings of this study are available from the corresponding author upon reasonable request.
