# Supplementary material for: Indirect Striatal Projection Neurons Drive a D2 Receptor‐Dependent Pathway to Dyskinesia and Dystonia
Source: Mov Disord. 2026 Apr 20;41(7):1690–703. doi: 10.1002/mds.70299 (PMC13388010; doi:10.1002/mds.70299)
Supplement: Supplementary file 3 — Data S1. Supporting Information. [file MDS-41-1690-s002.docx]

**Supplemental Methods**

*Animals and transgenic models*

Experiments were designed in accordance with PREPARE (Planning Research and Experimental Procedures on Animals: Recommendations for Excellence) ^1, 2^ and received ethical approval by the Malmö/Lund Ethics Committee on Animal Testing at Lund District Court. We used male and female mice of the C57BL/6 background, aged 8-12 weeks at the start of the experiments. To produce the conditional iSPN-D2R KO models we used two bacterial artificial chromosome (BAC) transgenic lines, namely B6.129S4 FVB-Drd2tm1.1Mrub/J mice (JAX020631, Jackson Labs) (herein referred to as Drd2^loxP/loxP^) and Adora2a-cre KG139Gsat/Mmucd mice (GENSAT) (herein A2aCre mice). This Adora2a-cre line has been shown to provide Cre-mediated recombination specifically in iSPNs ^3^. We also used the fluorescence reporter mice BAC-Drd1a-tdTomato (line B6.Cg-Tg(Drd1a-tdTomato)6Calak, JAX)^4^ and BAC-A2a-GFP mice (line Tg(Adora2a-cre)KG139Gsat/Mmucd, MMRRC) to identify dSPNs and iSPNs, respectively, in cellular verification studies.

*Adeno-associated viral vectors and delivery method*

All viral vectors were AAV2/5 pseudotyped and produced in our local core facility ([www.multipark.lu.se/infrastructures/cell-and-gene-therapy-core](http://www.multipark.lu.se/infrastructures/cell-and-gene-therapy-core)). To generate the unilateral iSPN-D2R KO model, we produced a vector coding for Cre under the proenkephalin promoter, which is iSPN-specific ^5^ (herein AAV-PENK-Cre vector). A flip-excision (flex) vector coding for green fluorescent protein (GFP) (AAV-flex-GFP) was used to verify the cellular specificity of Cre activity (cf. Figure 1). Stock vector preparations were diluted in phosphate buffered saline (PBS, pH 7.4) to a titer of 1.8*10^13^ (AAV-flex-GFP) or 1.1*10^13^ (AAV-PENK-Cre) genome copies/ml. Two injections of the AAV preparation (1 μL/injection) were delivered to the right dorsolateral striatum at the following coordinates (in mm, from bregma or the dural surface): (1) AP: + 1.0, L: - 2.1, DV: - 2.6; (2) AP: + 0.3, L: - 2.3, DV: - 2.6 (tooth bar: - 4.0), as in ^6^. When AAV injections were applied to 6-OHDA-lesioned animals, the AAV delivery preceded the 6-OHDA lesion by 3 weeks. In our initial knockout-validation studies, potential toxicity of the AAV transduction was ruled out by immunostaining striatal sections for the SPN-enriched cell marker, dopamine- and cyclic AMP-regulated phosphoprotein of 32 kDa (DARPP-32) (Cell Signaling rabbit anti DARPP-32 antibody #2302, 1:1000). The marker was uniformly expressed across the striatum, including AAV-transduced and non-transduced areas.

*Dopamine-denervating lesions*

Chronic striatal DA denervation was produced using 6-hydroxydopamine (6-OHDA-HCl, Sigma Aldrich, Stockholm, Sweden) as previously described ^7, 8^. Briefly, anesthetised mice (1.2-1.5% isoflurane/air mixture) received 1 µL of toxin solution (3.2 µg/µl 6-OHDA free base in 0.02% ascorbic acid-saline) in the right medial forebrain bundle (MFB) at the following coordinates: AP= -0,7; ML= 1,2; DV= -4,7 (tooth bar: - 4.0). Following surgery, mice received daily care for 2–3 weeks, as detailed in ^7, 8^ (which resulted in 100% postoperative survival), and a 3-week recovery period was allowed prior to any further experimental procedure. The dopaminergic denervation was verified in all animals using both the cylinder test of forelimb use asymmetry ^9^ and tyrosine hydroxylase (TH) immunohistochemistry. All the lesioned animals included in the study had > 85% loss of TH optical density (O.D.) in the striatum ipsilateral to the 6-OHDA infusion.

*Drugs, treatments, and the underlying rationale*

Dopaminergic drugs were administered using well characterised doses and treatment regimens ^7^. L-DOPA (L-3,4-dihydroxyphenylalanine methyl ester hydrochloride; Sigma-Aldrich AB, 6 mg/kg/day) was dissolved in physiological saline together with the peripheral DOPA decarboxylase inhibitor benserazide-HCl (Sigma-Aldrich AB; 12 mg/kg). The highly selective D2R full agonist sumanirole ^10^ (SUM) (Sumanirole maleate, Tocris, 4 mg/kg/day) and the partial D1R agonist SKF38393 (SKF) (Sigma-Aldrich AB, 3 mg/kg/day) were dissolved in physiological saline in a sonicating bath for approximately 10 minutes. Solutions were freshly prepared and injected intraperitoneally (i.p.) at a volume of 10 ml/kg body weight. Vehicle injections consisted of physiological saline solution. We opted to use SKF38393 instead of a full D1R agonist because SKF38393 better mimics the molecular and signaling response induced by L-DOPA in 6-OHDA lesioned rodents. Differently from SKF38393, full D1R agonists can induce ERK1/2 signaling activation, c-Fos and other LID-associated genes also in the intact striatum (both caudate-putamen and nucleus accumbens) ^11-13^. Moreover, behavioural and molecular responses to full D1R agonism are subject to rapid desensitization ^14^.

Subchronic drug treatment was administered by dosing each drug for 5 consecutive days, with a drug-free week between treatment blocks (i.e., no dosing from the Saturday after a treatment week until Monday of the week-after-next). Drugs were given in the following order: SUM, SKF, L-DOPA. This administration order was chosen to minimize the priming effect of one treatment relative to the next ones (L-DOPA having the most potent priming effect ^15-17^). The duration of subchronic drug treatment was chosen based on previous studies in mice with MFB lesions. When these mice receive daily injections of the same L-DOPA dose, the dyskinesia score per session plateaus as early as the second or third administration, with little or no further increase upon continued treatment ^5, 18, 19^ (see also Supplemental Results and Discussion II). The duration of the washout period (9 days) was chosen based on previous studies in 6-OHDA-lesioned rats, showing that the priming effect of subsequent acute drug challenges with DA agonists completely subsides within 10 days ^15^.

*Ratings of treatment-induced dyskinesia and dystonia*

For each drug treatment period, abnormal involuntary movements (AIMs) and dystonic features were assessed on treatment days 1, 3 and 5. Following the i.p. drug injection, mice were individually observed for 1 minute every 20 minutes for 3 hours to rate the severity of the following dyskinetic features: (i) axial AIMs (bending-twisting movements of the neck and upper body towards the side contralateral to the lesion); (ii) limb AIMs (circular or fluttering movements of the forelimb contralateral to the lesion); (iii) orofacial AIMs (empty jaw movements and twitching of facial muscles, occasionally accompanied by contralateral tongue protrusion). On each monitoring period, a 0-4 grading scale was applied to rate the severity of each AIM feature based on the proportion of observation time during which it was present ^7, 9^. The sum of axial, limb, and orolingual AIM scores provides a validated behavioural measure of LID in rodents ^20, 21^. The same time-based method was used to rate locomotor actions directed towards the side contralateral to the lesion (locomotive scores), which provide a generic index of motor activation during dopaminergic treatment in hemiparkinsonian rodent models ^18, 21^.

In addition to the on-line dyskinesia ratings, mice were video-recorded on each 1-min monitoring period to allow for an off-line evaluation of dystonic features using the method detailed in Andreoli et al. 2021 ^7^. Briefly, this method rates slow twisting movements and abnormal postures (sustained by active muscular activity) on a 0-3 severity scale based on the number of seconds during which the dystonic feature is present. We considered 6 topographic items, namely, trunk and neck (tr/ne), tail, hindlimbs and forelimbs contralateral and ipsilateral to the lesion ^7^. Scores from the hindlimbs and forelimbs of both sides were summed and referred to ‘HL’ and ‘FL’ dystonia.

*Behavioral parcellation analysis*

A behavioral parcellation analysis was carried out on the videos recorded at peak dyskinesia severity (40^th^ and 60^th^ min after drug injection) using the event-recording freeware JWatcher (<https://www.jwatcher.ucla.edu/>, V1.0), as in ^22^. We quantified the four most prevalent behavioral categories, that is: (i) *Contralateral Turn*, rotational movement of the head and body towards the side contralateral to the lesion (ipsilateral turning was not measured because it was totally absent on drug treatment); (ii) *Forward locomotion,* locomotor action with a straight body; (iii) *Grooming*, grooming sequence with bilateral involvement of the forelimbs; (iv) *Rearing,* vertical motions with forepaws placed on the walls of the cage. Note that, whereas *contralateral turning* can coexist with both AIMs and locomotive scores, the other three behavioral categories (*forward locomotion*, *grooming,* *rearing*) are normal behaviors, appearing at time points during which AIMs are absent. Only unequivocally classifiable video segments were included in the analysis. The investigator signaled the start and end of each behavioral episode by pressing a specific key, and results were computed as the proportion of active time (seconds) spent on each behavioral category.

*D2 receptor radioligand binding*

In our initial validation studies, a total of 21 unlesioned and drug-naïve mice were used to verify the striatal extent of D2R depletion using a radioligand binding method. Animals were sacrificed by decapitation, their brains were rapidly extracted and frozen on crushed dry-ice. Brains were cut on a cryostat at 16 µm thickness. Striatal sections were prewashed in 50 mM Tris-HCl buffer (containing 120 mM NaCl, 5 mM KCl, 2mM CaCl2 and 1 mM MgCl2; pH 7,4) for 20 minutes at room temperature. Slides were incubated for 1 hour in a buffer containing 4 nM of the radioactive D2 ligand [^3^H]raclopride (76 Ci/mmol) ^23^. To verify the ligand binding specificity, additional sections were co-incubated with non-radioactive raclopride (1 μM in the same buffer solution). After the incubation, slides were rinsed (1 min for 6 times) in ice-cold 50 mM Tris-HCl buffer (pH 7,4) and dipped in ice-cold distilled water. Slides were airdried and exposed to Kodak BioMax MR films for 10 weeks.

To measure the radioligand binding density, film autoradiographs (digitized using CanoScan8800F scanner) were visualised on the open-source image processing program Fiji (ImageJ softwares, NIH). The digitized images were converted to 8-bit grayscale, and optical density (O.D.) values were calibrated on the software internal standards. Measurements were performed bilaterally on 12 sections spanning the entire rostrocaudal extent of the caudate-putamen, and O.D. values were averaged across striatal sections after subtracting background values. In the bilateral iSPN-D2R KO model, [^3^H]raclopride binding density was expressed as a percentage of the values in the corresponding wildtype controls (see *Drd2*^+/+^ group in Fig. 1A-B). In the unilateral iSPN-D2R KO model, data were expressed as percentage of the values measured on the contralateral side in each animal.

*Immunohistochemistry*

Two to three weeks after completing the subchronic treatment sequence, mice were randomly allocated to receive a last injection of saline, sumanirole or L-DOPA. Thirty minutes after this last injection, mice were anesthetized with pentobarbital (600 mg/kg, i.p., Sanofi-Aventis) and perfused transcardially with 4% (w/v) paraformaldehyde in 0.1M phosphate buffer, pH 7.4. Brains were post-fixed overnight and then stored in PBS at 4°C until sectioning. Coronal sections (30 *µm*) were cut in PBS buffer using a vibratome (Leica, Germany) and stored at -20°C in a non-freezing solution containing 30% ethylene glycol and 30% glycerol in 0.1 M sodium phosphate buffer.

For brightfield immunohistochemistry, we used primary antibodies against Ser235/236-phosphorylated ribosomal protein S6 ^24^ (pS6) (Cell Signalling Technology, Antibody #2211 rabbit monoclonal recombinant, 1:400) and tyrosine hydroxylase (TH) (Pel Freez Biological, P40101, made in rabbit, 1:1000) Biotinylated secondary antibodies were from Vector Laboratories (goat anti-rabbit BA 1000, 1:400 for pS6 and 1:200 for TH ), followed by avidin-biotin peroxidase solution used according to the manufacturer´s instructions (ABC Elite Kit, Vector Laboratories). The final colour reaction was developed using 3,3’-diaminobenzidine (DAB) in 0.04% H_2_O_2_.

For confocal fluorescence microscopy, we used primary antibodies against Cre recombinase (Cell Signalling #15036, made in rabbit 1:500; used to assess the virally transduced region in the unilateral iSPN-D2R knockout model); choline acetyl transferase (ChAT) (Abcam AB144P, made in goat, 1:200, used to identify cholinergic interneurons); Red Fluorescent Protein (RFP) (Abcam ab185921, made in rabbit, 1:400, used to detect TdTomato, see Figure 1 G-H); GFP (Abcam, ab 290 made in rabbit or ab13970 made in chicken, 1:1000); pS6 (Cell Signalling Technology, Antibody #2211, made in rabbit, 1:100); parvalbumin (Synaptic Systems SySy 195 308, guinea pig monoclonal recombinant, 1:500); FoxP2 (AbCam ab1307, made in goat, 1:400),

The following secondary antibodies were used:

• Secondary antibodies from Jackson ImmunoResearch Laboratories: Cy5 donkey anti-goat (AB 2340415, 1:400); Cy3 donkey anti-rabbit (AB2307443, 1:400); Cy3 donkey anti-goat (AB2340411, 1:1000) ; Cy5 donkey anti-rabbit (AB2340607, 1:400).

• Secondary antibodies from Invitrogen: Alexa Flour 488 donkey anti-rabbit (A21206, 1:400); Alexa Fluor 488 goat anti-guinea pig (A11073 1:1000); Alexa Fluor 647 donkey anti-rabbit (A31573, 1:1000).

*Quantitative image analysis*

Tyrosine hydroxylase (TH)-immunostaining was measured on three striatal sections per animal spanning across the body of the caudate-putamen. Sections were digitized under a 20x objective on a Nikon Eclipse 80i microscope equipped with the Nikon DMS 1200F video camera. Optical density measurements were carried out as described in ^7, 9^ using the open-source image processing program Fiji (https://imagej.net/software/fiji/).

For cellular colocalization analyses, confocal pictures were acquired under a 20x or 40x objective (for dorsolateral striatum and GPe, respectively) on a Leica SP8 confocal laser scanning microscope, examining at least 9 sample areas per structure per animal (areas were distributed over 3 and 2 rostrocaudal levels for striatum and GPe, respectively). Confocal images (format 1024*1024 pixels) were acquired from one optical plane at 10-12 µm depth into the section. Counts of single- and double-labelled neurons were carried out using the cell-detection tool in the open-source software QuPath (version 0.5.1).

To count pS6-immunoreactive cells on bright-field microscopy, sections were scanned under a 40x objective (Slideview VS200, Evident). Images were converted to 8-bit grayscale, and pS6-positive cells were counted as single particles (Fiji multi-point tool), setting the same background threshold in all sample areas. Striatal pS6 cell counts were carried out on three coronal sections per animal (AP + 0.98 mm, + 0.38 mm, and + 0.14 mm from Bregma ^25^). On each section, cells were counted in three sample areas (0,18 mm^2^/area), representing dorso-central, dorso-lateral and ventro-lateral regions, and values from all sample areas were then averaged. Pallidal pS6 cell counts were carried out through the cross-sectional area of the GPe on two rostrocaudal levels per animal (AP - 0.46 mm, - 0.94 mm from Bregma ^25^).

*Statistical analyses*

Statistical analyses were performed using Prism 9 (GraphPad software). Output variables were compared between mouse genotypes and time points using repeated measures analysis of variance (ANOVA) or two-factor ANOVA, where appropriate. Pairwise post-hoc comparisons were carried out where appropriate using Tukey´s or Bonferroni´s test (both corrected for multiple comparisons). All main effects related to behavioural ratings (AIMs, locomotive or dystonia scores) were verified using non-parametric tests (Kruskal-Wallis or Mann-Whitney test depending on the type of comparison). The level of significance was set at α = 0.05.

**Literature citations**

1. Smith AJ, Clutton RE, Lilley E, Hansen KEA, Brattelid T. Improving animal research: PREPARE before you ARRIVE. BMJ 2018;360:k760.

2. Smith AJ, Clutton RE, Lilley E, Hansen KEA, Brattelid T. PREPARE: guidelines for planning animal research and testing. Lab Anim 2018;52(2):135-141.

3. Lemos JC, Friend DM, Kaplan AR, et al. Enhanced GABA Transmission Drives Bradykinesia Following Loss of Dopamine D2 Receptor Signaling. Neuron 2016;90(4):824-838.

4. Ade KK, Wan Y, Chen M, Gloss B, Calakos N. An Improved BAC Transgenic Fluorescent Reporter Line for Sensitive and Specific Identification of Striatonigral Medium Spiny Neurons. Front Syst Neurosci 2011;5:32.

5. Heiman M, Schaefer A, Gong S, et al. A translational profiling approach for the molecular characterization of CNS cell types. Cell 2008;135(4):738-748.

6. Alcacer C, Andreoli L, Sebastianutto I, Jakobsson J, Fieblinger T, Cenci MA. Chemogenetic stimulation of striatal projection neurons modulates responses to Parkinson's disease therapy. J Clin Invest 2017;127(2):720-734.

7. Andreoli L, Abbaszadeh M, Cao X, Cenci MA. Distinct patterns of dyskinetic and dystonic features following D1 or D2 receptor stimulation in a mouse model of parkinsonism. Neurobiol Dis 2021;157:105429.

8. Sebastianutto I, Maslava N, Hopkins CR, Cenci MA. Validation of an improved scale for rating l-DOPA-induced dyskinesia in the mouse and effects of specific dopamine receptor antagonists. Neurobiol Dis 2016;96:156-170.

9. Francardo V, Recchia A, Popovic N, Andersson D, Nissbrandt H, Cenci MA. Impact of the lesion procedure on the profiles of motor impairment and molecular responsiveness to L-DOPA in the 6-hydroxydopamine mouse model of Parkinson's disease. Neurobiol Dis 2011;42(3):327-340.

10. McCall RB, Lookingland KJ, Bedard PJ, Huff RM. Sumanirole, a highly dopamine D2-selective receptor agonist: in vitro and in vivo pharmacological characterization and efficacy in animal models of Parkinson's disease. J Pharmacol Exp Ther 2005;314(3):1248-1256.

11. Gerfen CR, Surmeier DJ. Modulation of striatal projection systems by dopamine. Annu Rev Neurosci 2011;34:441-466.

12. Wang JQ, McGinty JF. Scopolamine augments c-fos and zip/268 messenger RNA expression induced by the full D(1) dopamine receptor agonist SKF-82958 in the intact rat striatum. Neuroscience 1996;72(3):601-616.

13. Wang JQ, McGinty JF. The full D1 dopamine receptor agonist SKF-82958 induces neuropeptide mRNA in the normosensitive striatum of rats: regulation of D1/D2 interactions by muscarinic receptors. J Pharmacol Exp Ther 1997;281(2):972-982.

14. Asin KE, Bednarz L, Nikkel A, Perner R. Rotation and striatal c-fos expression after repeated, daily treatment with selective dopamine receptor agonists and levodopa. J Pharmacol Exp Ther 1995;273(3):1483-1490.

15. Morelli M, Fenu S, Garau L, Di Chiara G. Time and dose dependence of the 'priming' of the expression of dopamine receptor supersensitivity. Eur J Pharmacol 1989;162(2):329-335.

16. Nadjar A, Gerfen CR, Bezard E. Priming for l-dopa-induced dyskinesia in Parkinson's disease: a feature inherent to the treatment or the disease? Prog Neurobiol 2009;87(1):1-9.

17. Skovgard K, Barrientos SA, Petersson P, Halje P, Cenci MA. Distinctive Effects of D1 and D2 Receptor Agonists on Cortico-Basal Ganglia Oscillations in a Rodent Model of L-DOPA-Induced Dyskinesia. Neurotherapeutics 2023;20(1):304-324.

18. Fasano S, Bezard E, D'Antoni A, et al. Inhibition of Ras-guanine nucleotide-releasing factor 1 (Ras-GRF1) signaling in the striatum reverts motor symptoms associated with L-dopa-induced dyskinesia. Proc Natl Acad Sci U S A 2010;107(50):21824-21829.

19. Lundblad M, Picconi B, Lindgren H, Cenci MA. A model of L-DOPA-induced dyskinesia in 6-hydroxydopamine lesioned mice: relation to motor and cellular parameters of nigrostriatal function. Neurobiol Dis 2004;16(1):110-123.

20. Lundblad M, Andersson M, Winkler C, Kirik D, Wierup N, Cenci MA. Pharmacological validation of behavioural measures of akinesia and dyskinesia in a rat model of Parkinson's disease. Eur J Neurosci 2002;15(1):120-132.

21. Lundblad M, Usiello A, Carta M, Hakansson K, Fisone G, Cenci MA. Pharmacological validation of a mouse model of l-DOPA-induced dyskinesia. Exp Neurol 2005;194(1):66-75.

22. Wolfschlag M, Espa E, Skovgard K, Halje P, Cenci MA. Impulsive-compulsive behaviours and striatal neuroactivity in mildly parkinsonian rats under D2/3 agonist and L-DOPA treatment. NPJ Parkinsons Dis 2025;11(1):142.

23. Mansour A, Meador-Woodruff JH, Bunzow JR, Civelli O, Akil H, Watson SJ. Localization of dopamine D2 receptor mRNA and D1 and D2 receptor binding in the rat brain and pituitary: an in situ hybridization-receptor autoradiographic analysis. J Neurosci 1990;10(8):2587-2600.

24. Gangarossa G, Perroy J, Valjent E. Combinatorial topography and cell-type specific regulation of the ERK pathway by dopaminergic agonists in the mouse striatum. Brain Struct Funct 2013;218(2):405-419.

25. Paxinos G, Franklin KBJ. The mouse brain in stereotaxic coordinates. 2nd ed. San Diego: Academic Press, 2001.
