# Supplementary material for: Indirect Striatal Projection Neurons Drive a D2 Receptor‐Dependent Pathway to Dyskinesia and Dystonia
Source: Mov Disord. 2026 Apr 20;41(7):1690–703. doi: 10.1002/mds.70299 (PMC13388010; doi:10.1002/mds.70299)
Supplement: Supplementary file 4 — Figure S1. Motor phenotype in mice with bilateral iSPN‐D2R ablation before or after a 6‐OHDA lesion. Open‐field motions over 120 min and cylinder test were recorded under drug‐free conditions both before (A–F') and after (G–L') a 6‐OHDA lesion in the right MFB. Data show comparisons between mice with normal levels of D2R in iSPNs (Drd2+/+, n = 13) and hemizygous (Drd2KO+/−, n = 14) or homozygous iSPN‐D2R knockout mice (Drd2KO−/−, n = 13/12). (A, G) Percentage of right turns (ipsilateral to the side to be lesioned). (B, H) Cylinder test, percentage of contralateral forelimb use. (C, I) Sum of immobility episodes (≥3 s duration/episode) in the recording session. (D, J) Time course of rearing events and (D', J') sum of rearing events. (E, K) Time course of distance travelled in open‐field test and (E', K') sum of distance travelled during the test. (F, L) Time course of motion speed (max speed/bin) and (F', L') average max‐speed values in the recording session. (M) Motion traces during a 2‐hr exploration of the open field arena before and after the 6‐OHDA lesion. Yellow squares demarcate the center of the arena versus the periphery. (N) Ratio of time spent in the center versus the periphery (grey shade shows DA‐intact condition). Data are represented as mean ± SEM (line diagrams) or box and whiskers. Kruskal–Wallis test with Dunn's post‐hoc comparisons. *P < 0.05, **P < 0.01, ***P < 0.001 (vs. Drd2+/+). ^P < 0.05, ^^P < 0.01 (Drd2+/− vs. Drd2−/−) or Mann–Whitney U test. & P < 0.05 (vs. DA‐intact). Figure S2. Motor phenotype in mice with unilateral iSPN‐D2R ablation before or after a 6‐OHDA lesion. Open‐field motions over 120 min and cylinder test were recorded under drug‐free conditions both before (A–F') and after (G–L') a 6‐OHDA lesion in the right MFB. Data show comparisons between Drd2loxP‐/loxP‐ (AAV‐Drd2WT; n = 8) or Drd2loxP+/loxP+ (AAV‐Drd2KO; n = 12) mice injected with the AAV‐PENK‐Cre construct in the right dorsolateral striatum. (A, G) Percentage of right tu [file MDS-41-1690-s001.docx]

***Indirect striatal projection neurons drive a D2 receptor-dependent pathway to dyskinesia and dystonia***

**Supplemental Results**

**Table of Contents:**

Supplemental Results I: Motor phenotypes in the conditional iSPN-D2R KO models before and after the 6-OHDA lesion. page 2

*Bilateral iSPN-D2R knockout model* page 2

*Unilateral iSPN-D2R knockout model* page 3

Supplemental Discussion I page 4

Supplemental Results II: Evolution of dyskinesia scores over the drug treatments page 5

*Bilateral iSPN-D2R knockout model* page 5

*Unilateral iSPN-D2R knockout model* page 6

Supplemental Discussion II page 7

*Impact of the iSPN-D2R KO on the evolution of dyskinetic behaviors* page 7

*Further considerations about the significance of drug treatment duration*  page 8

Supplemental Results III: Dyskinesia and dystonia upon treatment with the D1 receptor agonist SKF38393 (detailed analysis from the last test session) page 10

**References**  page 11

**Supplemental Figures and legends**

Figure S1 page 13

Figure S2 page 15

Figure S3 page 17

Figure S4 page 19

Figure S5 page 21

Figure S6 page 23

**Supplemental Table**

Table S1 page 24

**Supplemental Results** **I.** **Motor phenotypes in the conditional iSPN-D2R KO models before and after the 6-OHDA lesion**

Prior to receiving any drug treatment, mice with conditional iSPN-D2R ablation and their corresponding wildtype controls were examined using open-field activity recordings and a test of forelimb use asymmetry (cylinder test) ^1, 2^. Open-field motions were monitored for 120 min in plexiglas boxes (40x40 cm in size) using ANY-maze video tracking (Stoelting). Turning movements were defined as 360° turns of the body axis. Immobile episodes were counted when the centroid of the animal´s body did not move for at least 3 consecutive seconds. In the bilateral iSPN-D2R KO model, mice were recorded under DA-intact conditions at the age of 8-12 weeks, and then again at 3 weeks after 6-OHDA lesion surgery. In the unilateral iSPN-D2R KO model, the DA-intact condition was assessed at 4 weeks post AAV-PENK-Cre injection, and mice were tested again 3 weeks after the 6-OHDA lesion.

*Bilateral iSPN-D2R knockout model*

Before the 6-OHDA lesion, the bilateral iSPN-D2R ablation did not cause any turning asymmetries (Fig. S1,A) nor forelimb use asymmetries in the cylinder test (Fig. S1,B). However, both homozygous and hemizygous iSPN-D2R ablations markedly increased the number of immobility episodes in the open-field recordings (Fig. S1,C, p < 0.01 vs Drd2^+/+^ controls for both KO groups). Homozygous Drd2KO^-/-^ mice, moreover, exhibited a marked reduction in the number of rearing events (Fig. S1,D-D´, p < 0.0001 vs Drd2^+/+^ controls), whereas the hemizygous Drd2KO^+/-^ animals did not differ significantly from control mice on this parameter. Additionally, both Drd2KO^+/-^ and Drd2KO^-/-^ mice showed a significant reduction in distance travelled, by 40% and 60%, respectively, compared with Drd2^+/+^ controls (Fig. S1,E-E´, p < 0.01 vs Drd2^+/+^ group in both KO genotypes). Homozygous Drd2KO^-/-^ mice also exhibited marked reductions in motion speed (Fig. S1,F-F’; p < 0.0001 vs Drd2^+/+^ group).

Animals were assessed again on the open-field and cylinder test 3 weeks after 6-OHDA lesion surgery. The unilateral 6-OHDA lesion produced a marked ipsilateral turning bias in both Drd2^+/+^ and Drd2KO^+/-^ mice (approx. 90% and 78% ipsilateral turns, respectively; Fig. S1,G). The turning bias was reduced or abolished in the homozygous Drd2KO^-/-^ mice, whose ipsilateral-turn median value was approx. 50% (Fig S1,G, p < 0.0001 vs Drd2^+/+^, p < 0.05 vs Drd2KO^+/-^). Unilateral striatal denervation is known to cause forelimb use asymmetry due to a reduced use of the contralateral forelimb in spontaneously initiated movements ^2^. Accordingly, 6-OHDA-lesioned mice of all three genotypes showed a lower use of the contralateral forelimb relative to the ipsilateral one in the cylinder test (Fig. S1,H). However, the effect of the lesion was more pronounced in Drd2^+/+^ and Drd2KO^+/-^ mice (Fig. S1,H, median value ~ 20% left paw use in both groups) compared to the homozygous Drd2KO^-/-^ animals (Fig. S1,H, median value ~ 30%, p < 0.05 vs Drd2^+/+^ controls). The above results indicate that a homozygous iSPN-D2R ablation significantly attenuates motor asymmetries induced by unilateral dopaminergic denervation.

In the post-lesion open-field activity recordings, the number of immobile episodes (Fig. S1,I) and rearing events were similarly low in all three groups (Fig. S1,J-J´). However, while Drd2KO^+/-^ and Drd2^+/+^ mice exhibited a marked decline in vertical activity relative to their pre-lesion values (cf. J-J´with D-D´), homozygous Drd2^-/-^ mice maintained approximately the same values (median total number ~ 200 rearing events both before and after the lesion, see Drd2KO^-/-^ group in J´ vs D´). These data indicate that homozygous ablation of iSPN-D2Rs had occluded the lesion-induced drop in rearing behavior.

After the 6-OHDA lesion, hemizygous Drd2KO^+/-^ mice and Drd2^+/+^ controls showed similar levels of distance travelled (Fig. S1,K-K´) and motion speed (Fig. S1,L-L´), with values clearly lower than before the lesion in each group (cf. K-K´ with E-E´, and L-L´ with F-F´). In homozygous Drd2KO^-/-^ mice, distance travelled and movement speed were similar to the two other groups´ only during the first 20 minutes of the test, but then rose from 30 min onwards until the end of the recording session (p < 0.05 for genotype effect and genotype-time interaction in Fig. S1,K,L). Accordingly, cumulative levels of distance travelled and speed values were significantly larger in homozygous Drd2KO^-/-^ mice relative to both hemizygous KO and control mice (Fig. S1,K´,L´). This paradoxical increase in horizontal activity was accompanied by an increased proportion of time spent in the center zone of the arena and a larger number of periphery-to-center entries compared to those measured before the lesion (Fig. S1,M-N).

*Unilateral iSPN-D2R knockout model*

Before the 6-OHDA lesion, the unilateral ablation of iSPN D2Rs had a marked effect on the animals´ turning behavior, with a median of 81% of turns being directed towards the virally transduced side in the AAV-Drd2KO group (Fig. S2,A, p < 0.001 vs AAV-Drd2WT). This ipsilateral turning bias mimics the effects of a unilateral DA-denervating lesion ^2^. In addition, the use of the forelimb contralateral to the virally transduced side was significantly reduced (Fig. S2,B, ~30% vs 50% contralateral forelimb use in AAV-Drd2KO vs AAV-Drd2WT mice, p < 0.001). These results indicate that unilateral iSPN-D2R in the dorsal striatum mimics the motor asymmetry caused by DA depletion. The time course analysis of vertical activity showed a significant effect of genotype, as the number of rearing events was lower in the AAV-Drd2KO compared to the AAV-Drd2WT group at most time points (Fig. S2,D). However, the total number of rearing events during the session did not differ significantly between the groups (Fig. S2,D´), nor did the number of immobility episodes (Fig. S2,C). No differences were found between the AAV-Drd2KO and AAV-Drd2WT animals in the analysis of distance travelled and motion speed (Fig. S2,E-E´ and F-F´).

After the 6-OHDA lesion, mice of both genotypes exhibited a prominent ipsilateral turning bias, with 86% and 95% ipsilateral turns (median values) recorded in the AAV-Drd2WT and AAV-Drd2KO groups, respectively (Fig. S2,G, p = 0.048). In the cylinder test, both groups showed a marked and comparable degree of forelimb use asymmetry, with only 20-25% of paw placements being performed with the forelimb contralateral to the lesion (Fig. S2,H). These results show that a DA-denervating lesion ipsilateral to the virally transduced side obliterated the difference between AAV-Drd2KO animals and controls on a measure of lateralized paw use. The number of rearing events did not differ significantly between AAV-Drd2KO and AAV-Drd2WT (Fig. S2,J-J´). In both groups, the number of rearing events was lower than that measured before the lesion, although the decline from pre-lesion values was more pronounced in the AAV-Drd2WT animals (cf. J-J´ with D-D´ in Fig. S2). In the analysis of distance travelled and motion speed, AAV-Drd2KO mice exhibited higher activity levels than controls (Fig. S2,K-L, p < 0.05 for genotype effect in the time course analysis; Fig. S2,K´-L´, p < 0.05 for AAV-Drd2KO vs AAV-Drd2WT in the cumulative analysis). In addition, the time spent in the center zone and the number of periphery-to-center entries were significantly increased in the AAV-Drd2KO after the lesion (Fig. S2,M-N, p < 0.05 vs DA-intact).

**Supplemental Discussion I.**

In keeping with previous studies ^3, 4^, we found that bilateral ablation of iSPN D2Rs significantly reduces the levels of horizontal and vertical activity in the open field. In contrast, mice with unilateral iSPN-D2R ablation (AAV-Drd2KO) (still having intact dopaminergic neurons) did not show any clear sign of hypokinesia or bradykinesia in their open-field motions. Indeed, distance travelled, speed, vertical exploratory behavior (rearings) and immobility periods were similar in AAV-Drd2KO and the AAV-injected *Drd2*^WT^ controls. Ipsilateral turning (towards the KO side) and reduced ipsilateral forelimb use were the only clear phenotype caused by the unilateral iSPN-D2R deletion in DA-intact mice. Interestingly, these motor abnormalities resemble the effects of unilateral DA-denervating lesions in intact mice ^2^. Thus, a deletion of iSPN-D2Rs restricted to one side of the dorsal striatum recapitulates the motor asymmetries associated with unilateral dopaminergic degeneration, but does not result in a clear reduction of whole-body movements during open field exploration. The latter effect is associated with an enhanced GABAergic transmission in the striatum ^4^, and apparently requires a striatum-wide loss of iSPN D2R signaling, as seen in our bilateral KO model.

In the post-lesion test sessions, mice with homozygous iSPN-D2R ablation (both Drd2KO^-/-^ and AAV-Drd2KO mice) exhibited reduced levels of vertical exploration, while their horizontal activity either remained unaltered or showed augmentation. The paradoxical increase in horizontal activity became apparent after the first 10-15 minutes of the recording session. In the initial 15-minute bin, the KO animals (both Drd2KO^-/-^ and AAV-Drd2KO) exhibited low levels of distance travelled and motion speed similarly to their WT controls. The augmentation of horizontal activity in DA-denervated mice lacking iSPN D2Rs most likely reflects a failure to habituate to the test environment, and suggests an impairment in iSPN-related plasticity processes ^5^. It is noteworthy that 6-OHDA-lesioned KO mice from both genetic models (Drd2KO^-/-^ and AAV-Drd2KO) showed increased exploratory behavior towards the center of the arena, a region that rodents normally avoid as potentially dangerous. Therefore, an increased centre/periphery ratio of open-field motions suggests a reduced sensitivity to negative behavioral outcomes, a function that is controlled by iSPNs ^6^.

**Supplemental Results II: Evolution of dyskinesia scores over the drug treatments**

As explained in the Methods section and illustrated in Fig. 1,I-J mice were treated sequentially with sumanirole (4 mg/kg), SKF38393 (SKF, 3 mg/kg), and L-DOPA (6 mg/kg) at previously characterized doses ^1^. Each drug was given once a day for 5 consecutive days, leaving a drug-free week between consecutive treatments. Behavioral tests were carried out on days 1, 3, and 5 in each drug treatment period.

*Bilateral iSPN-D2R knockout model*

*• Sumanirole.* The evolution of sumanirole-induced AIM scores over the treatment period is shown in Fig. S3,A-D. The sum of axial, limb and orofacial AIM scores per session differed markedly between genotypes and days (p < 0.01 and p < 0.0001 for day and genotype effect, respectively, p > 0.05 for day*genotype interaction) (Fig. S3,A). The strong effect of genotype was driven by a reduced AIM severity in the hemizygous Drd2KO^+/-^ group and a total lack of AIMs in Drd2KO^-/-^ mice (p < 0.05 for Drd2KO^+/-^ vs Drd2^+/+^ through all test days). The interaction between test day and genotype was driven by an increase in AIM scores between day 1 and day 5 in the Drd2^+/+^ group (p < 0.05 for day 1 vs day 5). Time-action curves of sumanirole-induced dyskinesia from each test session are shown in Fig. S3,B-D. On each test day, the AIM score per monitoring period differed significantly between post-injection time point and genotypes (p < 0.0001 for the effects of time, genotype, and time*genotype interaction). At no time point after sumanirole dosing did Drd2KO^-/-^ mice show any dyskinesia (p < 0.01 for Drd2KO^-/-^ vs Drd2^+/+^ at 40-140 min post dosing on day 1; p < 0.05 for the same group comparison at 20-100 min post-dosing on days 3, p < 0.05 for the same group comparison at 20-120 min post-dosing on day 5). Hemizygous Drd2KO^+/-^ mice did not differ significantly from the Drd2KO^-/-^ group on the first treatment day (Fig. S3,B) but showed an increase in AIM scores on days 3-5 (Fig S3,C-D) though not reaching the same levels seen in wild-type controls (p < 0.05 for Drd2KO^+/-^ vs Drd2^+/+^ at 60-80 min on day 5).

*• SKF38393 (SKF).* SKF-induced AIM scores are shown in Fig. S3,E-H. The sum of axial, limb and orofacial AIM scores per session did not differ significantly between test days or genotypes (p > 0.05 for the effects of day, genotype, and day*genotype interaction, Fig. S3,E). Time-action curves of SKF-induced AIM scores were similar between test days, with the highest scores recorded at 20-40 min post injection, followed by a rapid decline and a complete return to baseline by 120 min (Fig. S3,F-H; p < 0.001 for time effect, p > 0.05 for the effects of genotype and time*genotype interaction on each test day).

*• L-DOPA.* The evolution of L-DOPA-induced AIM scores across test sessions is shown in Fig. S3,I-L. The sum of axial, limb and orofacial AIM scores per session differed significantly between treatment days and genotypes (p < 0.05 and p < 0.01 for day and genotype effect, respectively, p > 0.05 for day*genotype interaction) (Fig. S3,I). In both the Drd2^+/+^ and the hemizygous Drd2KO^+/-^ mice, the sum of AIM score tended to decline from day 1 to day 3-5, although the corresponding between-day comparisons did not reach significance (Fig. S3,I). Drd2KO^-/-^ mice showed a transient increase in AIM scores between day 1 and day 3 (p< 0.05), which did not persist until day 5 (p > 0.05 vs day 1). Time-action curves of L-DOPA-induced dyskinesia from each test session are shown in Fig. S3,J-L. On the first treatment day, the overall effects of both monitoring period and genotype were clearcut (p<0.01 for both time effect and time*genotype interaction, p < 0.05 for genotype effect). Indeed, Drd2KO^-/-^ mice showed a conspicuous reduction in both dyskinesia peak severity and duration compared to the wild-type control group (Fig. S3,J, p < 0.05 for Drd2KO^-/-^ vs Drd2^+/+^ at 20-60 and 100 min). On day 3, the effects of genotype and time-genotype interaction did not reach significance. This was most likely due to lower peak dyskinesia scores in the wild-type group on the third day of treatment (cf. 20-60 min score for Drd^+/+^ group in Figs. S3,K vs J), so that the AIM curve in this group became almost overlapping with that in hemizygous Drd2KO^+/-^ mice (cf. Drd2^+/+^ and Drd2KO^+/-^ groups in Fig. S3,K), whereas peak dyskinesia scores remained lower in Drd2KO^-/-^ mice. The effect of genotype reached significance again on the fifth day of treatment, with a clear reduction in AIM scores in Drd2KO^-/-^ mice compared to Drd2^+/+^ controls (Fig. S3,L, the same data are reported in Fig 2I and the corresponding Results section).

*Unilateral iSPN-D2R knockout model*

*• Sumanirole.* The evolution of sumanirole-induced AIM scores is shown in Fig. S4,A-D. The sum of axial, limb and orofacial AIM scores per session differed significantly between treatment days and genotypes (p < 0.0001 for the effects of day, genotype, and day*genotype interaction) (Fig. S4,A). The strong effect of genotype was driven by a total lack of AIMs in the AAV-Drd2KO group from day 1 to day 5 (p < 0.001 AAV-Drd2KO vs AAV-Drd2WT through all test days). (Fig. S4,A). The effect of test day was driven by an increase in AIM scores between day 1 and day 3 in the wild-type control mice (p < 0.001 for day 1 vs. both day 3 and 5 in AAV-Drd2WT group). Time-action curves of sumanirole-induced dyskinesia from each test session are shown in Fig. S4,B-D. On each test day, the AIM score per monitoring period differed significantly between time points and genotypes (p < 0.0001 for the effects of time point, genotype, and time*genotype interaction). No dyskinesia was present at any time point after sumanirole administration in the AAV-Drd2KO mice (p < 0.05 AAV-Drd2KO at 40-120 min on day 1, 20-140 min on day 3, 20-100 min on day 5).

*• SKF38393 (SKF).* SKF-induced AIM scores are shown in Fig. S4,E-H. The evolution of axial, limb and orofacial AIM scores per session differed between genotypes (p < 0.001 for the effects of genotype and day*genotype interaction, p < 0.01 for day) (Fig. S4,E). These effects were driven by unexpectedly high AIM scores in the AAV-Drd2WT group on the first test session (p < 0.001 vs AAV-Drd2KO on day 1). The very high AIM scores measured in the control group on day 1 do not conform to the typical effect of SKF38393 in this mouse model (cf. SKF data in Fig. S3, and see ^1^). AIM scores in the control group fell to the expected range on days 3-5, where no significant difference was detected between AAV-Drd2WT and AAV-Drd2KO mice (Fig. Fig. S4,E, p < 0.01 for day 5 vs day 1 in the AAV-Drd2WT group). Time-action curves of SKF-induced dyskinesia from each test session are shown in Fig. S4,F-H. The AIM score per monitoring period differed significantly between post-injection time point and genotype only at day 1 (Fig. S4,F, p < 0.0001 for the effects of time, genotype, and time*genotype interaction; p < 0.05 for vs AAV-Drd2WT vs AAV-Drd2KO at 20–100 minutes). On day 3 and 5, AIM time curves did not differ significantly between the experimental groups (p > 0.05 for genotype and time*genotype interaction, p < 0.001 for time point).

*• L-DOPA.* The evolution of L-DOPA-induced AIM scores is shown in Fig. S4,I-L. A comparison of the AIM scores per session revealed a highly significant effect of genotype across test days (Fig. S4,I, p < 0.0001 for genotype and day*genotype interaction, p > 0.05 for day). Time-action curves of L-DOPA-induced AIMs from each test session are shown in Fig. S4,J-L. On each day, the AIM score per monitoring period differed significantly between post-injection time points (p < 0.0001 for time effect) and genotypes (p < 0.001 for both genotype and time*genotype interaction; Figs. S4,J-L). During both the peak phase and the decline phase of the time-action curve, AIM scores were markedly lower in the AAV-Drd2KO group compared to wild-type controls (p < 0.05 at 40-60 and 100 minutes on day 1, at 20-80 minutes on day 3 and on day 5).

**Supplemental Discussion II.**

*Impact of the iSPN-D2R KO on the evolution of dyskinetic behaviors*

The results presented in Figs. S3 and S4 supplement the behavioral data from the fifth day of sumanirole and L-DOPA treatment that are presented and discussed in the main body of the manuscript. The analysis of AIMs data across all test sessions corroborates the significant effect of iSPN-D2R deletion in abolishing or reducing dyskinesias elicited by D2R agonism or L-DOPA, respectively, with minor or inconsistent effects seen on SKF-induced AIMs. Five consecutive days of L-DOPA treatment is a shorter treatment duration than that used in previous publications studying the impact of D2R KO on LID. In previous studies, 6-OHDA-lesioned mice were treated with a fixed dose of L-DOPA for 10 days (15 mg/kg/day) ^3^ or 18 days (25 mg/kg/day) ^7^. These treatments were given to mice with intrastriatal 6-OHDA lesions, which require larger L-DOPA dosages than MFB- lesioned mice to develop equivalent levels of dyskinesia ^8^. Another study used unlesioned mice having a selective knockout of the long D2R isoform (the most abundant isoform of D2R in the brain), which received 20 mg/kg/day L-DOPA for 21 days ^9^. In the three studies here mentioned, the effect of D2R ablation on L-DOPA-induced AIMs did not differ in either direction or magnitude from the fourth day of treatment onwards. Therefore, it is very unlikely that the different outcome of D2R ablation reported in these previous studies (dyskinesia augmentation or no effect) compared to ours (dyskinesia reduction) depended on different durations of L-DOPA treatment. It is more likely that the different outcomes reflect the use of different genetic models. In the three mentioned studies ^3, 7, 9^, the Drd2 genetic deletion started from embryonic age. This is important because a constitutive genetic ablation of D2R signaling causes prominent compensatory mechanisms that do not appear to occur in mice rendered D2R-deficient as adults ^10^. Florio and colleagues produced a conditional iSPN-D2R KO model by mating Drd2^loxP-loxP^ mice with a constitutive Drd1-Cre transgenic line ^3^. The rationale behind this strategy is that the D1R gene is expressed in the majority of SPN precursors during embryonic age ^11, 12^, thus enabling the Cre-lox recombination to occur in immature iSPNs before these neurons silence their expression of Drd1 (which occurs mainly postnatally ^12, 13^). The choice of crossing Drd2^loxP-loxP^ mice with a Drd1-Cre rather than Drd2-Cre line is plausibly based on the intention to avoid Cre-recombination in striatal cholinergic interneurons, which do express both the Drd2 and the Adora2a gene in the adult striatum. Luckily, the transgenic *Adora2*-Cre mouse in our study exhibits negligible Cre recombination in cholinergic interneurons (see Fig. 1 and corresponding results narrative, see also ^4^), a favorable feature most likely reflecting an effect of the transgenic insertion site in this particular mouse line.

The specific functional consequences of ablating D2R signaling during development have not been established. However, interesting hints can be found in the comprehensive transcriptomics study published by Florio and colleagues ^3^, showing extensive adaptations of the striatal cellular network involving multiple molecular pathways. Gene expression data obtained from their iSPN-D2R KO model before the lesion showed a significant upregulation of multiple genes that are strongly expressed in both iSPNs and dSPNs and regulate their responses to DA/L-DOPA, including some dSPN-specific genes (e.g. muscarinic M4 receptor). At 60 min post L-DOPA administration, the majority of differentially expressed gene in KO vs wild-type animals were associated with the control of transcription ^3^. This pattern is indicative of a strong response to L-DOPA in dSPNs, which are transcriptionally very active up to 2 hours after L-DOPA administration, whereas iSPNs are not (reviewed in ^14^). Upregulated genes included some whose expression in dSPNs has been found to correlated with dyskinesia severity in earlier literature. Taken together, the data suggest that the augmented LID severity in this specific KO model most likely reflected an enhanced sensitization of dSPN signaling during the treatment.

*Further considerations about the significance of drug treatment duration*

We used a treatment regimen consisting of 5 consecutive daily drug injections based on our previous studies. In MFB-lesioned mice, LID scores plateau by the second or third day of treatment with the same L-DOPA dose, with little or no further increase thereafter ^8, 14, 15^. To induce progressive increases in dyskinesia severity in this lesion model, it is necessary to apply incremental L‑DOPA dosing regimens ^3, 8, 14, 15^. In line with our previous studies, the data shown in Figs. S3-S4 (panels A-E-I) confirm that AIM scores did not differ significantly between day 3 and 5 in any group or treatment. The results from the 5^th^ day of treatment can therefore be considered to represent the steady-state phase of dyskinesia development in this animal model. With longer treatment durations, changes in the shape of the L-DOPA time-action curve (earlier peak-steeper decline) may bring about a reduction rather than an increase in the AIM score per session over repeated administrations ^16^. Accordingly, we observed trends for both L-DOPA- and SKF-induced AIM scores to decrease between day 1 and day 5 due to the fact that the AIMs peaked earlier and declined faster upon continued treatment. An equivalent trend was not evident upon treatment with sumanirole, suggesting that the observed shortening of the AIMs curve likely depends on D1R-dependent signaling adaptations. Supporting this suggestion, earlier studies measuring drug-induced rotations in 6-OHDA-lesioned rats reported a significant response attenuation over repeated treatment with selective D1R but not D2R agonists ^17^.

In many rodent studies examining LID-related plasticity, chronic treatment is defined as a period of 8-10 daily L-DOPA injections ^14, 15, 18-21^. This treatment duration is indeed sufficient to induce transcriptional changes indicative of synaptic and structural plasticity, associated with sustained striatal remodeling (reviewed in ^22^). In a transcriptomics study in mice comparing the effects of acute, subchronic (5 days) and chronic (10 days) L-DOPA treatment, the subchronic and chronic groups were eventually pooled together, suggesting a lack of substantial differences between the two treatment periods ^20^. It is nevertheless conceivable that certain plastic changes associated with L-DOPA treatment develop only after several weeks of drug administration, for example, changes affecting the function of striatal cholinergic interneurons ^23^. Further studies examining the impact of iSPN-D2R ablation over longer periods of L-DOPA treatment are therefore warranted.

**Supplemental Results** **III.** **Dyskinesia and dystonia upon treatment with the D1 receptor agonist SKF38393 (detailed analysis from the last test session)**

We have previously shown that the partial D1R agonist SKF38393 induces mild dyskinesia of short duration, mainly consisting of limb and orofacial AIMs ^1^. The same effect pattern was produced by SKF38393 (herein SKF) in mice with normal D2R expression, i.e., the Drd2^+/+^ group in Fig. S5,A-C and the AAV-Drd2WT group in Fig. S5,I-K.

In the model with bilateral iSPN-D2R ablation (Fig. S5,A-H), SKF-induced axial, limb and oroligual AIM scores did not differ significantly between the three genotypes (p > 0.05 for genotype effect and genotype-time interaction in Fig. S5,A; p > 0.05 for total AIM scores in Fig. S5,B; p > 0.05 for genotype and genotype-AIM subtype interaction in Fig. S5,C). The analysis of other behaviors and locomotive scores revealed a significant genotype effect (Fig. S5,D-F; p < 0.01 for genotype), which was accounted for by larger locomotive scores and contralateral turning in the homozygous Drd2KO^-/-^ mice compared to the other two groups (Fig. S5,D, F; p < 0.05 for Drd2KO^-/-^ vs Drd2^+/+^). As expected based on previous studies ^1^, treatment with SKF induced very mild dystonic features, predominantly consisting of sustained tail dorsiflexion (Fig. S5,G-H). The dystonia subscore analysis revealed an effect of genotype, which depended on a increased expression of tail dystonia in the homozygous Drd2KO^-/-^ mice in the absence of any other topographic subtype (Fig. S5,H, p < 0.01 for dystonia subscore-genotype interaction, p=0.089 for genotype effect).

In the model with unilateral iSPN-D2R ablation (AAV-Drd2KO in Fig. S5,I-P), axial, limb and orofacial AIM scores tended to be lower than controls at 20-60 min post SKF administration (Fig. S5,I, p = 0.056 for genotype effect, p < 0.05 for genotype-time interaction). However, the total AIM score per session did not differ significantly between the two genotypes (Fig. S5,J) (which is in keeping with the bilateral iSPN-D2R KO model, cf. Fig. S5,B). A trend towards a genotype effect (p=0.056) was detected in the AIM subscore analysis, plausibly driven by the total absence of axial AIMs in the AAV-Drd2KO group (Fig. S5,K). No genotype effect was detected in other behaviors or locomotive scores (Fig. S5,L-N). Treatment with SKF induced the expected low levels of dystonia in both genotypes, and the only dystonic feature detected in AAV-Drd2KO animals was tail dystonia (Fig. S5,O-P).

**References**

1. Andreoli L, Abbaszadeh M, Cao X, Cenci MA. Distinct patterns of dyskinetic and dystonic features following D1 or D2 receptor stimulation in a mouse model of parkinsonism. Neurobiol Dis 2021;157:105429.

2. Francardo V, Recchia A, Popovic N, Andersson D, Nissbrandt H, Cenci MA. Impact of the lesion procedure on the profiles of motor impairment and molecular responsiveness to L-DOPA in the 6-hydroxydopamine mouse model of Parkinson's disease. Neurobiol Dis 2011;42(3):327-340.

3. Florio E, Serra M, Lewis RG, et al. D2R signaling in striatal spiny neurons modulates L-DOPA induced dyskinesia. iScience 2022;25(10):105263.

4. Lemos JC, Friend DM, Kaplan AR, et al. Enhanced GABA Transmission Drives Bradykinesia Following Loss of Dopamine D2 Receptor Signaling. Neuron 2016;90(4):824-838.

5. Durieux PF, Schiffmann SN, de Kerchove d'Exaerde A. Differential regulation of motor control and response to dopaminergic drugs by D1R and D2R neurons in distinct dorsal striatum subregions. EMBO J 2012;31(3):640-653.

6. Kravitz AV, Kreitzer AC. Striatal mechanisms underlying movement, reinforcement, and punishment. Physiology (Bethesda) 2012;27(3):167-177.

7. Darmopil S, Martin AB, De Diego IR, Ares S, Moratalla R. Genetic inactivation of dopamine D1 but not D2 receptors inhibits L-DOPA-induced dyskinesia and histone activation. Biol Psychiatry 2009;66(6):603-613.

8. Lundblad M, Picconi B, Lindgren H, Cenci MA. A model of L-DOPA-induced dyskinesia in 6-hydroxydopamine lesioned mice: relation to motor and cellular parameters of nigrostriatal function. Neurobiol Dis 2004;16(1):110-123.

9. Li L, Cheng L, Wang Y. Differential roles of two isoforms of dopamine D2 receptors in l-dopa-induced abnormal involuntary movements in mice. Neuroreport 2021;32(7):555-561.

10. Bello EP, Casas-Cordero R, Galinanes GL, et al. Inducible ablation of dopamine D2 receptors in adult mice impairs locomotion, motor skill learning and leads to severe parkinsonism. Mol Psychiatry 2017;22(4):595-604.

11. Anzalone A, Lizardi-Ortiz JE, Ramos M, et al. Dual control of dopamine synthesis and release by presynaptic and postsynaptic dopamine D2 receptors. J Neurosci 2012;32(26):9023-9034.

12. Thibault D, Loustalot F, Fortin GM, Bourque MJ, Trudeau LE. Evaluation of D1 and D2 dopamine receptor segregation in the developing striatum using BAC transgenic mice. PLoS One 2013;8(7):e67219.

13. Novak G, Fan T, O'Dowd BF, George SR. Striatal development involves a switch in gene expression networks, followed by a myelination event: implications for neuropsychiatric disease. Synapse 2013;67(4):179-188.

14. Heiman M, Schaefer A, Gong S, et al. A translational profiling approach for the molecular characterization of CNS cell types. Cell 2008;135(4):738-748.

15. Fasano S, Bezard E, D'Antoni A, et al. Inhibition of Ras-guanine nucleotide-releasing factor 1 (Ras-GRF1) signaling in the striatum reverts motor symptoms associated with L-dopa-induced dyskinesia. Proc Natl Acad Sci U S A 2010;107(50):21824-21829.

16. Lundblad M, Usiello A, Carta M, Hakansson K, Fisone G, Cenci MA. Pharmacological validation of a mouse model of l-DOPA-induced dyskinesia. Exp Neurol 2005;194(1):66-75.

17. Asin KE, Bednarz L, Nikkel A, Perner R. Rotation and striatal c-fos expression after repeated, daily treatment with selective dopamine receptor agonists and levodopa. J Pharmacol Exp Ther 1995;273(3):1483-1490.

18. Dyavar SR, Potts LF, Beck G, et al. Transcriptomic approach predicts a major role for transforming growth factor beta type 1 pathway in L-Dopa-induced dyskinesia in parkinsonian rats. Genes Brain Behav 2020;19(8):e12690.

19. El Atifi-Borel M, Buggia-Prevot V, Platet N, Benabid AL, Berger F, Sgambato-Faure V. De novo and long-term l-Dopa induce both common and distinct striatal gene profiles in the hemiparkinsonian rat. Neurobiol Dis 2009;34(2):340-350.

20. Figge DA, Amaral HO, Crim J, Cowell RM, Standaert DG, Eskow Jaunarajs KL. Differential Activation States of Direct Pathway Striatal Output Neurons during l-DOPA-Induced Dyskinesia Development. J Neurosci 2024;44(26).

21. Santini E, Heiman M, Greengard P, Valjent E, Fisone G. Inhibition of mTOR signaling in Parkinson's disease prevents L-DOPA-induced dyskinesia. Sci Signal 2009;2(80):ra36.

22. Cenci MA, Ferrario JE. Molecular mechanisms of L-DOPA-induced dyskinesia. In: Moratalla R, Murer MG, eds. Handbook of Parkinson´s disease mechanisms: Elsevier B.V., 2025:235-249.

23. Ding Y, Won L, Britt JP, Lim SA, McGehee DS, Kang UJ. Enhanced striatal cholinergic neuronal activity mediates L-DOPA-induced dyskinesia in parkinsonian mice. Proc Natl Acad Sci U S A 2011;108(2):840-845.

**Figure S1**

**
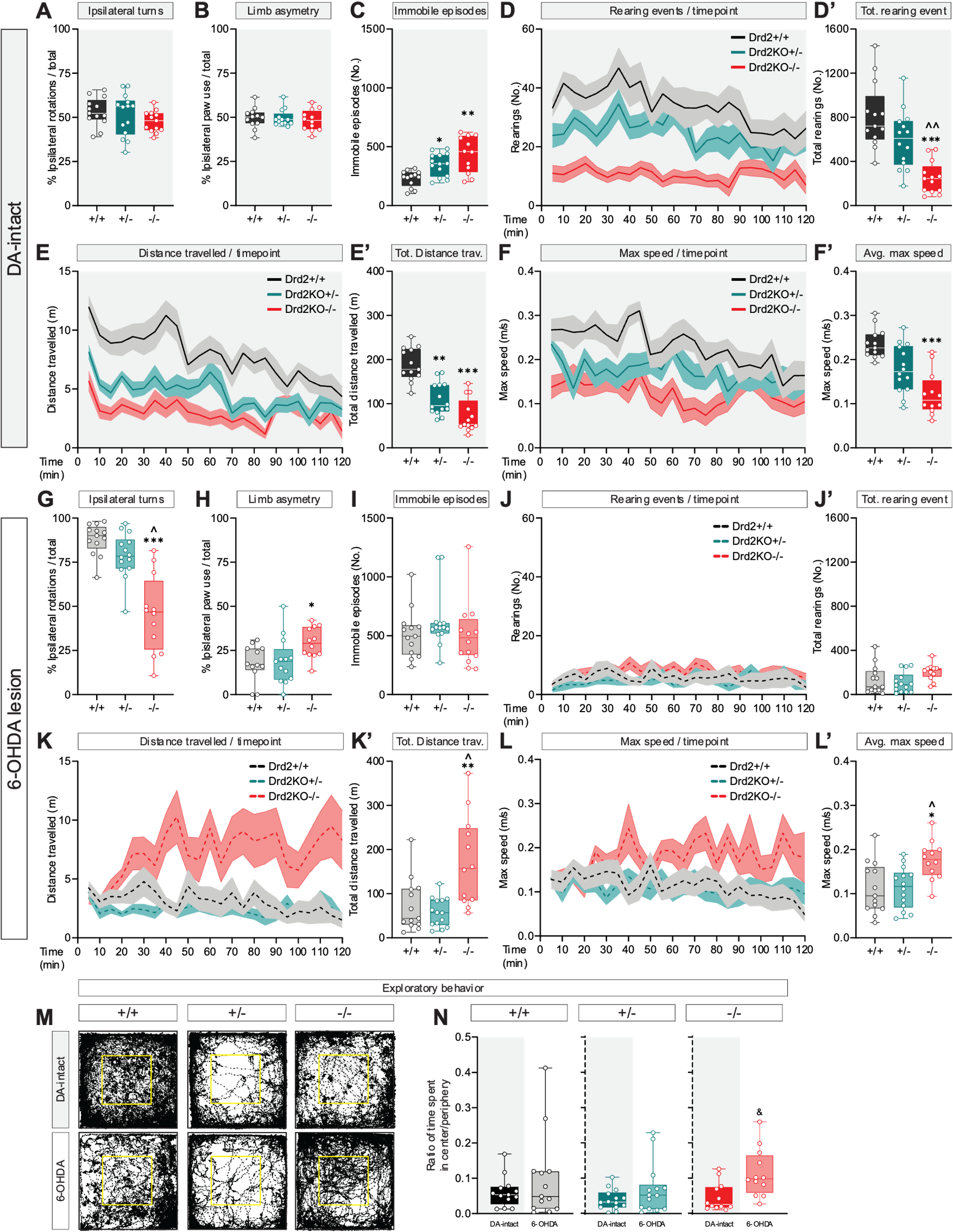
**

**Figure S1. Motor phenotype in mice with bilateral iSPN-D2R ablation before or after a 6-OHDA lesion.** Open-field motions over 120 minutes and cylinder test were recorded under drug-free conditions both before **(A-F’)** and after **(G-L’)** a 6-OHDA lesion in the right MFB. Data show comparisons between mice with normal levels of D2R in iSPNs (Drd2^+/+^, n=13) and hemizygous (Drd2KO^+/-^, n=14) or homozygous iSPN-D2R knockout mice (Drd2KO^-/-^, n=13/12). **(A, G)** Percentage of right turns (ipsilateral to the side to be lesioned). **(B, H)** Cylinder test - percentage of left forelimb use. **(C, I)** Sum of immobility episodes (≥3 sec duration/episode) in the recording session. **(D, J)** Time course of rearing events and **(D’, J’)** sum of rearing events. **(E, K)** Time course of distance travelled in open-field test and **(E’, K’)** sum of distance travelled during the test. **(F, L)** Time course of motion speed (max speed/ bin) and **(F’, L’)** average max-speed values in the recording session. **(M)** Motion traces during a 2-hour exploration of the open field arena before and after the 6-OHDA lesion. Yellow squares demarcate the center of the arena versus the periphery. (**N**) Ratio of time spent in the center vs the periphery (grey shade shows DA-intact condition *Data is represented as Mean ± SEM (line diagrams) or box and whiskers. Kruskal-Wallis test with Dunn’s post hoc comparisons. p* < 0.05, ** < 0.01, *** < 0.001 (vs Drd2^+/+^). p^ < 0.05, ^^ < 0.01 (Drd2^+/-^ vs Drd2^-/-^) or Mann-Whitney U test. p& < 0.05 (vs DA-intact)*

**Figure S2**

**
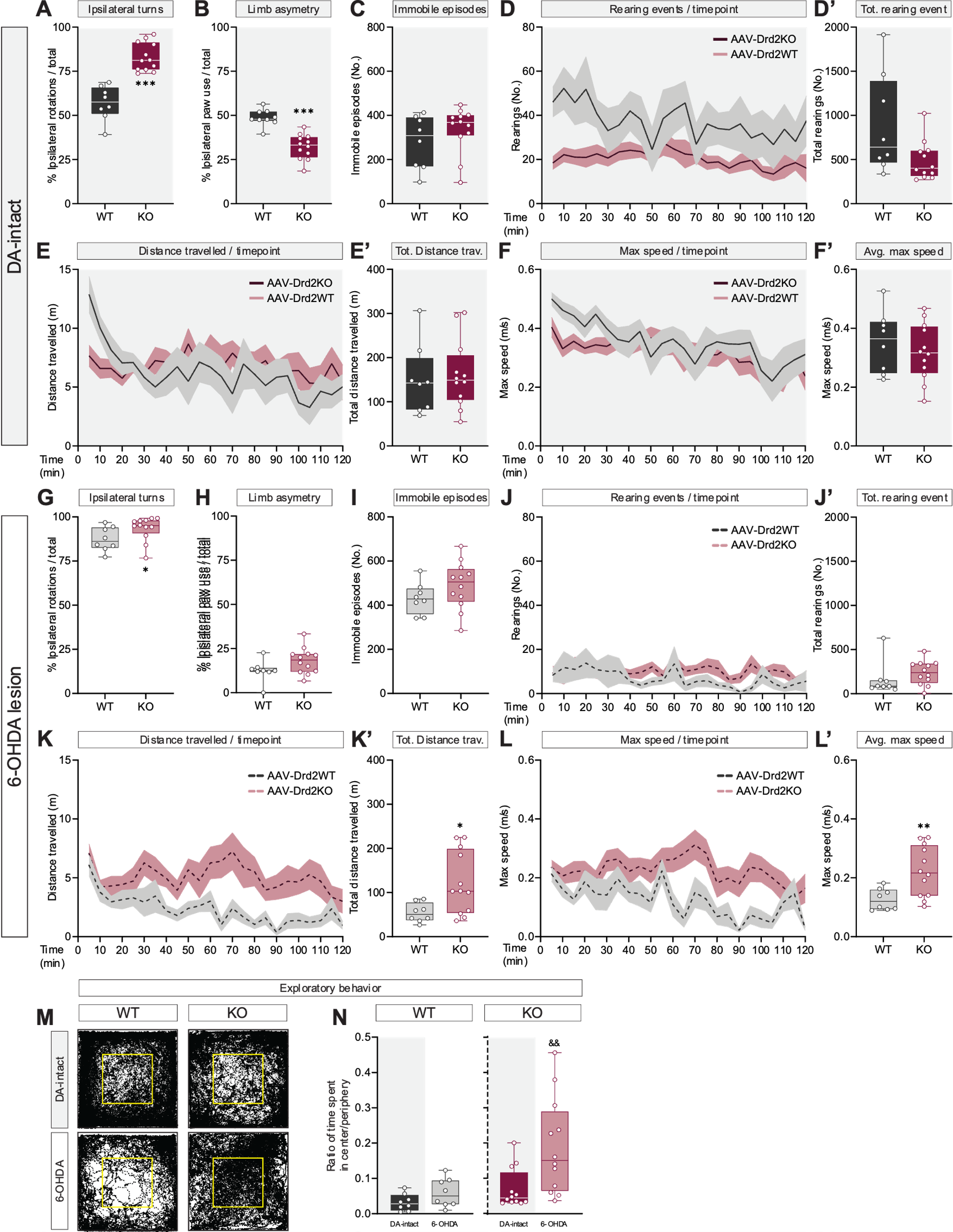
**

**Figure S2. Motor phenotype in mice with unilateral iSPN-D2R ablation before or after a 6-OHDA lesion.** Open-field motions over 120 minutes and cylinder test were recorded under drug-free conditions both before **(A-F’)** and after **(G-L’)** a 6-OHDA lesion in the right MFB. Data show comparisons between Drd2^loxP-/loxP-^ (AAV-Drd2WT; n=8) or Drd2^loxP+/loxP+^ (AAV-Drd2KO; n=12) mice injected with the AAV-PENK-Cre construct in the right dorsolateral striatum. **(A, G)** Percentage of right turns (ipsilateral to the side to be lesioned). **(B, H)** Cylinder test - percentage of left forelimb use. **(C, I)** Sum of immobility episodes (≥3 sec duration/episode) in the recording session. **(D, J)** Time course of rearing events and **(D’, J’)** sum of rearing events. **(E, K)** Time course of distance travelled in the open-field test and **(E’, K’)** sum of distance travelled during the test. **(F, L)** Time course of motion speed, expressed as max speed/recording bin and **(F’, L’)** average max-speed values in the recording session. **(M)** Motion traces during a 2-hour exploration of the open field arena both before and after a 6-OHDA lesion. Yellow squares demarcate the center of the arena versus the periphery. The ratio of time spent in the center vs the periphery of the arena is reported in panel **(N)** where the DA-intact condition is indicated with the grey shaded area. *Data is represented as Mean ± SEM (line diagrams) or box and whiskers. Mann-Whitney U test. p* < 0.05, ** < 0.01, *** < 0.001 (WT vs KO). p&& < 0.01 (vs DA-intact)*

**Figure S3**

**
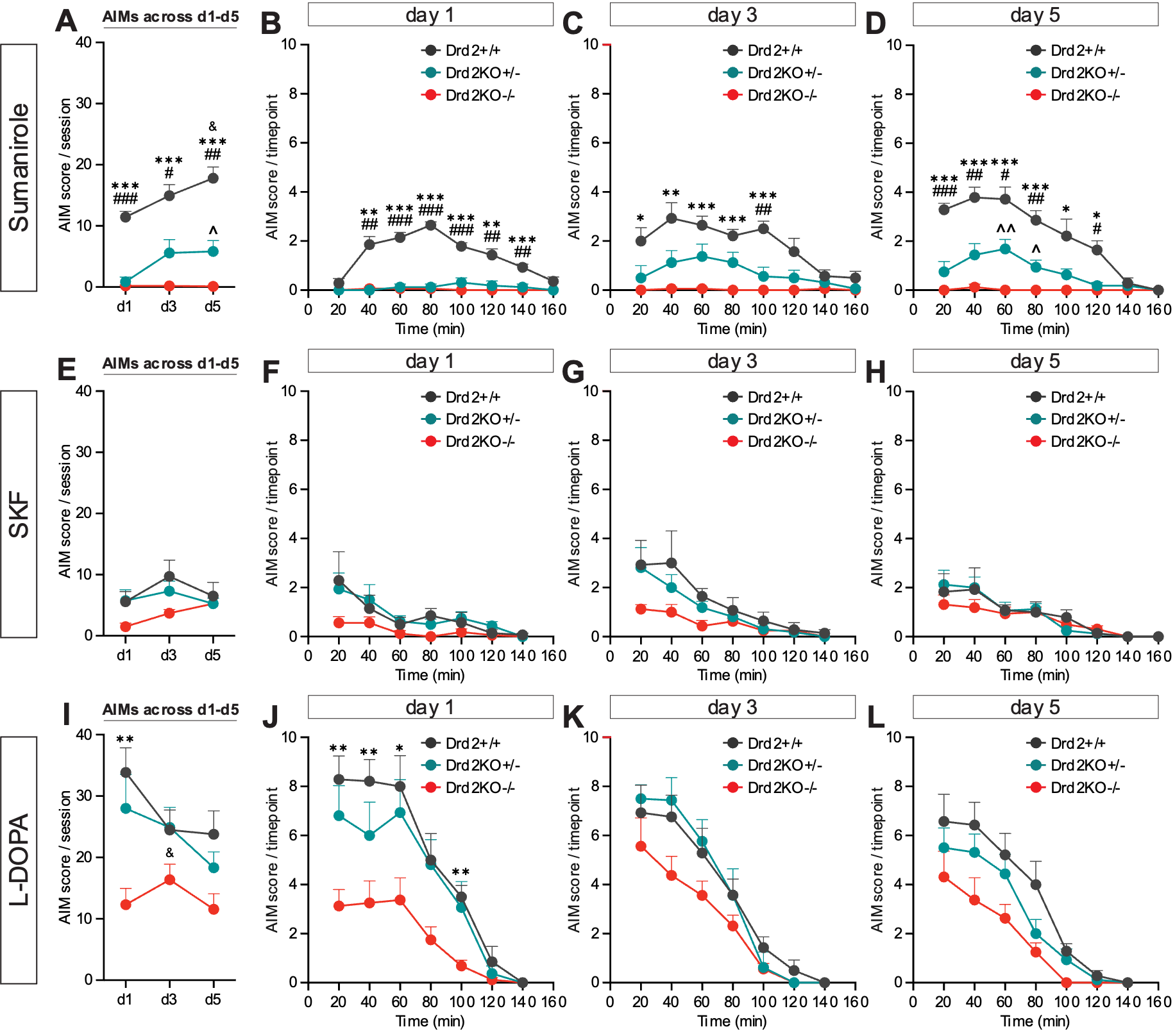
**

**Figure S3. Evolution of dyskinesia scores over the drug treatments in mice with bilateral ablation of iSPN D2Rs.** Animals correspond to figure 2 in the main manuscript. Data are reported over day 1, 3 and 5 of treatment with the D2R-class agonist sumanirole (4mg/kg) **(A-D),** the D1-class agonist SKF38393 (3.0 mg/kg) **(E-H)** and L-DOPA (6.0 mg/kg) **(I-L)**. **(A)** Sum of AIM scores per session over day 1, 3 and 5 of sumanirole treatment. *Two-way RM ANOVA; Genotype: F_(2, 20)_ 72.95, p < 0.001; Day: F_(1.391, 27.82)_ 7.49, p = 0.0058; Interaction: F_(2.782, 27.82)_ 2.23, p = 0.1108. Tukey’s post hoc comparisons: p*** < 0.001 vs Drd2^-/-^, p# < 0.05, p## < 0.01 and p### < 0.001 vs Drd2^+/-^, p ^ < 0.05 Drd2^+/-^ vs Drd2^-/-^.* **(B)** Time course of axial, limb and orofacial AIM scores (sum per monitoring period) on day 1 after sumanirole injection. *Two-way RM ANOVA; Genotype: F_(2, 20)_ 82.22, p < 0.0001; Day: F_(3.815, 76.30)_ 24.04, p < 0.0001; Interaction: F_(7.630, 76.30)_ 18.01, p < 0.0001. Tukey’s post hoc comparisons: p** < 0.01 and p ***< 0.001 vs Drd2^-/-^, p## < 0.01 and p### < 0.001 vs Drd2^+/-^.* **(C)** Time course of axial, limb and orofacial AIM scores (sum per monitoring period) on day 3 after sumanirole injection. *Two-way RM ANOVA; Genotype: F_(2, 20)_ 20.25, p < 0.0001; Day: F_(4.102, 82.03)_ 8.234, p < 0.0001; Interaction: F_(8.203, 82.03)_ 3.144, p = 0.0035. Tukey’s post hoc comparisons: p* < 0.05, p** < 0.01 and p ***< 0.001 vs Drd2^-/-^, p## < 0.01 vs Drd2^+/-^.* **(D)** Time course of axial, limb and orofacial AIM scores (sum per monitoring period) on day 5 after sumanirole injection. *Two-way RM ANOVA; Genotype: F_(2, 20)_ 36.96, p < 0.0001; Day: F_(2.392, 47.85)_ 27.09, p < 0.0001; Interaction: F_(4.785, 47.85)_ 10.49, p < 0.0001. Tukey’s post hoc comparisons: p* < 0.05 and p ***< 0.001 vs Drd2^-/-^, p# < 0.05, p## < 0.01 and p### < 0.001 vs Drd2^+/^.* **(E)** Sum of AIM scores per session over day 1, 3 and 5 of SKF treatment. *Two-way RM ANOVA; Genotype: F_(2, 20)_ 2.750, p = 0.001; Day: F_(1.530, 30.60)_ 3.542, p = 0.0525; Interaction: F_(3.060, 30.60)_ 1.462, p = 0.2440.* **(F)** Time course of axial, limb and orofacial AIM scores (sum per monitoring period) on day 1 after SKF injection. *Two-way RM ANOVA; Genotype: F_(2, 20)_ 2.933, p = 0.0764; Day: F_(2.167, 43.34)_ 8. 684, p = 0.0006; Interaction: F_(4.334, 43.34)_ 0.9642, p = 0.4417.* **(G)** Time course of axial, limb and orofacial AIM scores (sum per monitoring period) on day 3 after SKF injection. *Two-way RM ANOVA; Genotype: F_(2, 20)_ 3.001, p = 0.0725; Day: F_(1.839, 36.78)_ 15.69, p < 0.0001; Interaction: F_(3.678, 36.78)_ 1.309, p = 0.2854.* **(H)** Time course of axial, limb and orofacial AIM scores (sum per monitoring period) on day 5 after SKF injection. *Two-way RM ANOVA; Genotype: F_(2, 20)_ 0.3268, p = 0.7250; Day: F_(2.391, 47.47)_ 19.24, p < 0.0001; Interaction: F_(14, 139)_ 0.7019, p = 0.7696.* **(I)** Sum of AIM scores per session over day 1, 3 and 5 of L-DOPA treatment. *Two-way RM ANOVA; Genotype: F_(2, 20)_ 7.683, p = 0.0030; Day: F_(1.599, 31.98)_ 4.386, p = 0.0278; Interaction: F_(3.198, 31.98)_ 1.687, p = 0.1871. Tukey’s post hoc comparisons: p** < 0.01 vs Drd2^-/-^; p & < 0.05 Drd2^-/-^ vs day 1.* **(J)** Time course of axial, limb and orofacial AIM scores (sum per monitoring period) on day 1 after L-DOPA injection. *Two-way RM ANOVA; Genotype: F_(2, 20)_ 6.848, p = 0.0054; Day: F_(2.570, 51.40)_ 58.43, p < 0.0001; Interaction: F_(5.140, 51.40)_ 3.474, p = 0.0083. Tukey’s post hoc comparisons: p* < 0.05 and p** < 0.01 vs Drd2^-/-^.* **(K)** Time course of axial, limb and orofacial AIM scores (sum per monitoring period) on day 3 after L-DOPA injection. *Two-way RM ANOVA; Genotype: F_(2, 20)_ 3.078, p = 0.0683; Day: F_(3.557, 71.14)_ 74.26, p < 0.0001; Interaction: F_(7.114, 71.14)_ 1.297, p = 0.2640.* **(L)** Time course of axial, limb and orofacial AIM scores (sum per monitoring period) on day 5 after L-DOPA injection. *Two-way RM ANOVA; Genotype: F_(2, 20)_ 4.189, p = 0.0302; Day: F_(2.023, 40.47)_ 63.13, p < 0.0001; Interaction: F_(12, 120)_ 1.626, p = 0.0930.*

**Figure S4**

**
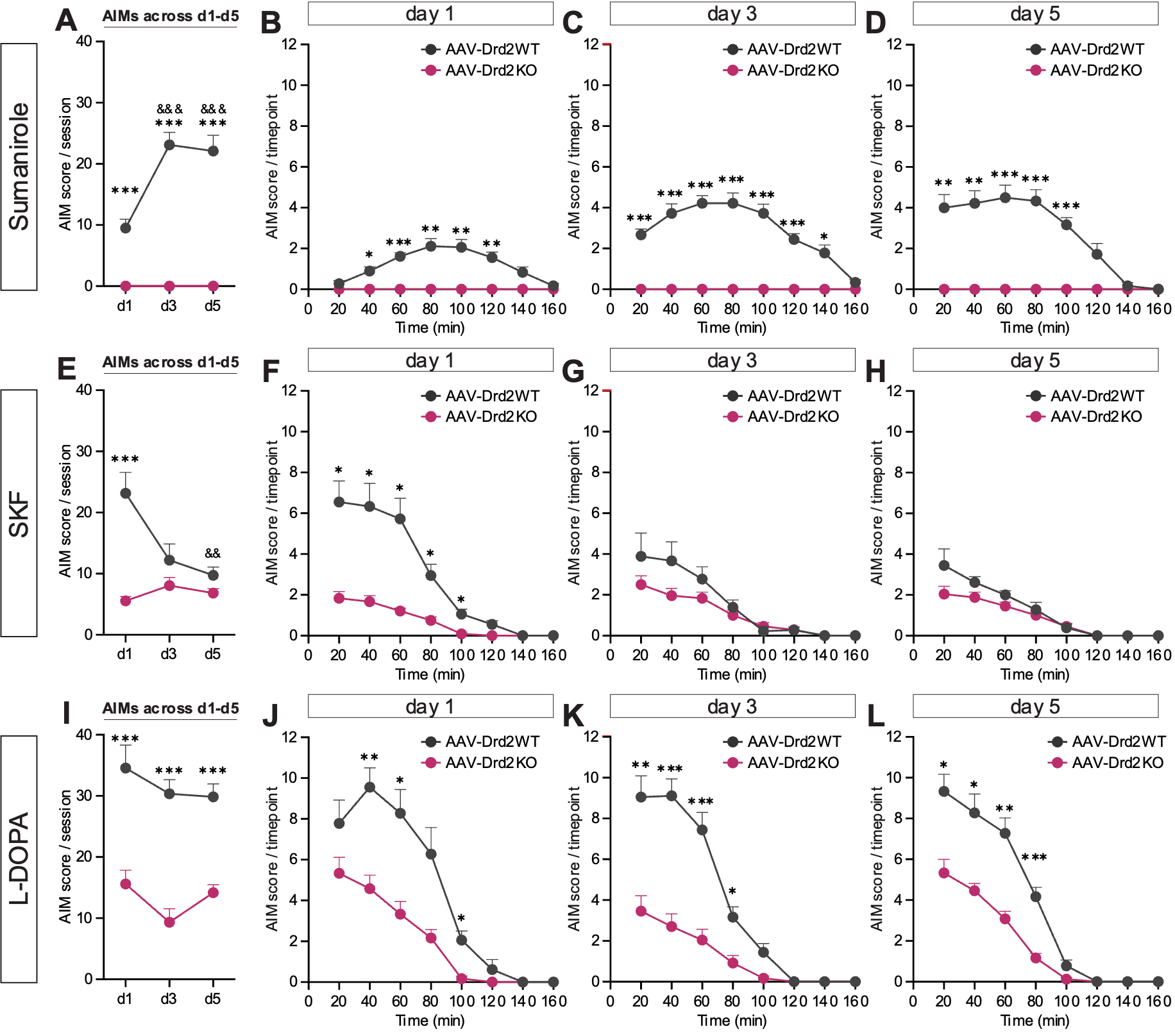
**

**Figure S4. Evolution of dyskinesia scores over the drug treatments in mice with unilateral ablation of iSPN D2Rs.** Animals correspond to figure 3 in the main manuscript. Data are reported over day 1, 3 and 5 of treatment with the D2R-class agonist sumanirole (4mg/kg) **(A-D),** the D1-class agonist SKF38393 (3.0 mg/kg) **(E-H)** and L-DOPA (6.0 mg/kg) **(I-L)**. **(A)** Sum of AIM scores per session over day 1, 3 and 5 of sumanirole treatment. *Two-way RM ANOVA; Genotype: F_(1, 19)_ 170.4, p < 0.0001; Day: F_(1.783, 33.88)_ 31.64, p < 0.0001; Interaction: F_(1.783, 33.88)_ 31.64, p < 0.0001. Tukey’s post hoc comparisons: p*** < 0.001 vs Drd2^+/+^, p&&& < 0.001, Drd2^+/+^ vs day 1of the same genotype.* **(B)** Time course of axial, limb and orofacial AIM scores (sum per monitoring period) on day 1 after sumanirole injection. *Two-way RM ANOVA; Genotype: F_(1, 19)_ 58.79, p < 0.0001; Day: F_(3.360, 63.84)_ 19.17, p < 0.0001; Interaction: F_(3.360, 63.84)_ 19.17, p < 0.0001. Tukey’s post hoc comparisons: p* < 0.05, p** < 0.01 and p ***< 0.001 vs Drd2^-/-^.* ***(*C)** Time course of axial, limb and orofacial AIM scores (sum per monitoring period) on day 3 after sumanirole injection. *Two-way RM ANOVA; Genotype: F_1, 19)_ 172.0, p < 0.0001; Day: F_(3.970, 75.42)_ 28.27, p < 0.0001; Interaction F_(3.970, 75.42)_ 28.27, p < 0.0001. Tukey’s post hoc comparisons: p* < 0.05 and p ***< 0.001 vs Drd2^-/-^.* **(D)** Time course of axial, limb and orofacial AIM scores (sum per monitoring period) on day 5 after sumanirole injection. *Two-way RM ANOVA; Genotype: F_(1, 19)_ 100.7, p < 0.0001; Day: F_(2. 697, 51.24)_ 30.70, p < 0.0001; F_(2. 697, 51.24)_ 30.70, p < 0.0001. Tukey’s post hoc comparisons: p** < 0.01 and p ***< 0.001 vs Drd2^-/-^.* **(E)** Sum of AIM scores per session over day 1, 3 and 5 of SKF treatment. *Two-way RM ANOVA; Genotype: F_(1, 19)_ 23.58, p = 0.0001; Day: F_(1.494, 28.38)_ 7.547, p = 0.0048; Interaction: F_(1.494, 28.38)_ 12.96, p = 0.0003. p ***< 0.001 vs Drd2^-/-^, p&& < 0.01, Drd2^+/+^ vs day 1of the same genotype.* **(F)** Time course of axial, limb and orofacial AIM scores (sum per monitoring period) on day 1 after SKF injection. *Two-way RM ANOVA; Genotype: F_(1, 19)_ 33.57, p < 0.0001; Day: F_(2.390, 45.41)_ 48.89, p < 0.0001; Interaction: F_(2.390, 45.41)_ 16. 37, p < 0.0001. p* < 0.05 vs Drd2^-/-^.* **(G)** Time course of axial, limb and orofacial AIM scores (sum per monitoring period) on day 3 after SKF injection. *Two-way RM ANOVA; Genotype: F_(1, 19)_ 2.294, p = 0.1463; Day: F_(1.885, 35.82)_ 29.13, p < 0.0001; Interaction: F_(1.885, 35.82)_ 2.137, p = 0.1353.* **(H)** Time course of axial, limb and orofacial AIM scores (sum per monitoring period) on day 5 after SKF injection. *Two-way RM ANOVA; Genotype: F_(1, 19)_ 4.114, p = 0.0568; Day: F_(1.994, 37.88)_ 42.62, p < 0.0001; Interaction: F_(1.994, 37.88)_ 2.217, p = 0.1230.* **(I)** Sum of AIM scores per session over day 1, 3 and 5 of L-DOPA treatment. *Two-way RM ANOVA; Genotype: F_(1, 19)_ 60.15, p < 0.0001; Day: F_(1.426, 27.10)_ 3.439, p = 0.0609; Interaction: F_(1.426, 27.10)_ 0.900, p = 0.3866. Tukey’s post hoc comparisons: p*** < 0.01 vs Drd2^-/-^.* **(J)** Time course of axial, limb and orofacial AIM scores (sum per monitoring period) on day 1 after L-DOPA injection. *Two-way RM ANOVA; Genotype: F_(1, 19)_ 20.91, p = 0.0002; Day: F_(2.632, 50.00)_ 63.52, p < 0.0001; Interaction: F_(2.632, 50.00)_ 7.319, p = 0.0006. Tukey’s post hoc comparisons: p* < 0.05 and p** < 0.01 vs Drd2^-/-^.* **(K)** Time course of axial, limb and orofacial AIM scores (sum per monitoring period) on day 3 after L-DOPA injection. *Two-way RM ANOVA; Genotype: F_(1, 19)_ 42.69, p < 0.0001; Day: F_(2.318, 44.05)_ 77.00, p < 0.0001; Interaction: F_(2.318, 44.05)_ 19.56, p < 0.0001. p* < 0.05, p** < 0.01 and p ***< 0.001 vs Drd2^-/-^.* **(L)** Time course of axial, limb and orofacial AIM scores (sum per monitoring period) on day 5 after L-DOPA injection. *Two-way RM ANOVA; Genotype: F_(1, 19)_ 43.95, p < 0.0001; Day: F_(2.224, 42.26)_ 136.1, p < 0.0001; Interaction: F_(2.224, 42.26)_ 13.81, p < 0.0001. p* < 0.05, p** < 0.01 and p ***< 0.001 vs Drd2^-/-^.*

**Figure S5**

**
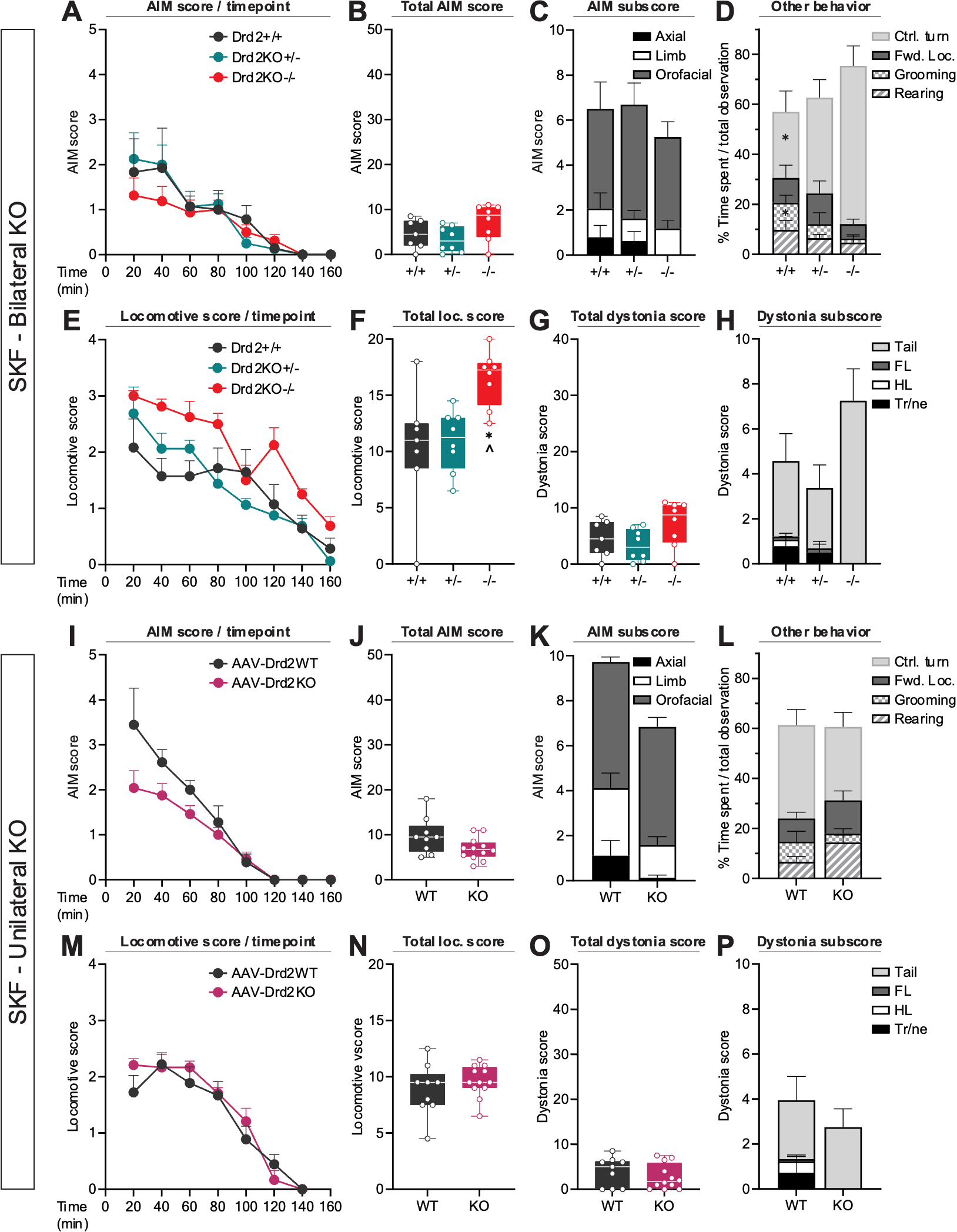
**

**Figure S5. Dyskinetic and dystonic features induced by a D1 receptor agonist are not significantly attenuated by the ablation of iSPN D2Rs.** Data are from the last treatment day with the D1R-class agonist SKF38393 (3.0 mg/kg) for the bilateral **(A-H)** and unilateral **(I-P)** iSPN-D2R knockout models. **(A)** Time course of axial, limb and orofacial AIM scores (sum per monitoring period). *Two-way RM ANOVA; Genotype: F_(2, 20)_ 0.33, p = 0.33; Interaction: F_(14, 139)_ 0.70, p = 0.7019.* **(B)** Sum of axial, limb and orofacial AIM scores in the corresponding test session. *Kruskal-Wallis test; KW(genotype) = 0.18, p = 0.9122.* **(C)** Breakdown of the AIM scores into the three dyskinesia subtypes, represented using different bar fillings (see caption). *Two-way RM ANOVA; Genotype: F_(2, 20)_ 0.28, p = 0.7612; Interaction: F_(2.6, 26.0)_ 0.50, p = 0.6588.* **(D)** Other behavior (Contralateral rotation, forward locomotion, grooming, and rearing) at the peak of dyskinesia (40 & 60 min after drug injection) as observed with event-recording software JWatcher. *Two-way RM ANOVA; Genotype: F_(2, 20)_ 4.56, P < 0.05; Interaction: F_(2.9, 29.2)_ 4.10, p < 0.05. Tukey’s post hoc comparisons: p* < 0.05 (vs -/-)* **(E)** Locomotive scores recorded during the corresponding test sessions. *Two-way RM ANOVA; Genotype: F_(2, 20)_ 5.94, p < 0.01; Interaction: F_(8.5, 84.0)_ 1.81, p = 0.0823.* **(F)** Sum of locomotor scores in the corresponding test session. *Kruskal-Wallis test; KW(genotype) = 10.03, p<0.01.* *Dunn’s post hoc comparisons: p* < 0.05 (vs Drd2^+/+^), p ^ < 0.05 (Drd2^+/-^ vs Drd2^-/-^)* **(G)** Total dystonia scores in the corresponding test session (sum of dystonia scores for trunk/neck, contralateral hindlimb, ipsilateral hindlimb, contralateral forelimb, ipsilateral forelimb, and tail). *Kruskal-Wallis test; KW(genotype) = 4.54, p = 0.1031.* **(H)** Breakdown of the dystonia scores into the four topographic subtypes, represented using different bar fillings (see caption). For this representation, the scores recorded from hindlimbs and forelimbs on both sides were summed together and named HL and FL, respectively. *Two-way RM ANOVA; Genotype: F_(2, 20)_ 2.73, p = 0.0893; Interaction: F_(6, 60)_ 4.08, p < 0.01.* **(I)** Time course of axial, limb and orofacial AIM scores (sum per monitoring period). *Two-way RM ANOVA; Genotype: F_(1, 19)_ 4.11, p = 0.0568; Interaction: F_(8, 152)_ 2.22, p < 0.05.* **(J)** Sum of axial, limb and orofacial AIM scores in the corresponding test session. *Mann-Whitney U test; p = 0.1061.* **(K)** Breakdown of the AIM scores into the three dyskinesia subtypes, represented using different bar fillings (see caption). *Two-way RM ANOVA; Genotype: F_(1, 19)_ 4.11, p = 0.0568; Interaction: F_(2, 38)_ 1.49, p = 0.2382.* **(L)** Other behavior (Contralateral rotation, forward locomotion, grooming, and rearing) at the peak of dyskinesia (40 & 60 min after drug injection) as observed with event-recording software JWatcher. *Two-way RM ANOVA; Genotype: F_(1, 18)_ 0.027, P = 0.8720; Interaction: F_(1.8, 31.9)_ 1.28, P = 0.2891.* **(M)** Locomotive scores recorded during the corresponding test sessions. *Two-way RM ANOVA; Genotype: F_(1, 19)_ 0.95, p = 0.3423; Interaction: F_(6, 114)_ 1.01, p = 0.4242.* **(N)** Sum of locomotor scores in the corresponding test session. *Mann-Whitney U test; p = 0.4037.* **(O)** Total dystonia scores in the corresponding test session (sum of dystonia scores for trunk/neck, contralateral hindlimb, ipsilateral hindlimb, contralateral forelimb, ipsilateral forelimb, and tail). *Mann-Whitney U test; p = 0.6343.* **(P)** Breakdown of the dystonia scores into the four topographic subtypes, represented using different bar fillings (see caption). For this representation, the scores recorded from hindlimbs and forelimbs on both sides were summed together and named HL and FL, respectively. *Two-way RM ANOVA; Genotype: F_(1, 19)_ 0.82, p = 0.3778; Interaction: F_(3, 57)_ 0.30, p = 0.8266. Data is represented as Mean ± SEM or box and whiskers.*

**Figure S6**

**
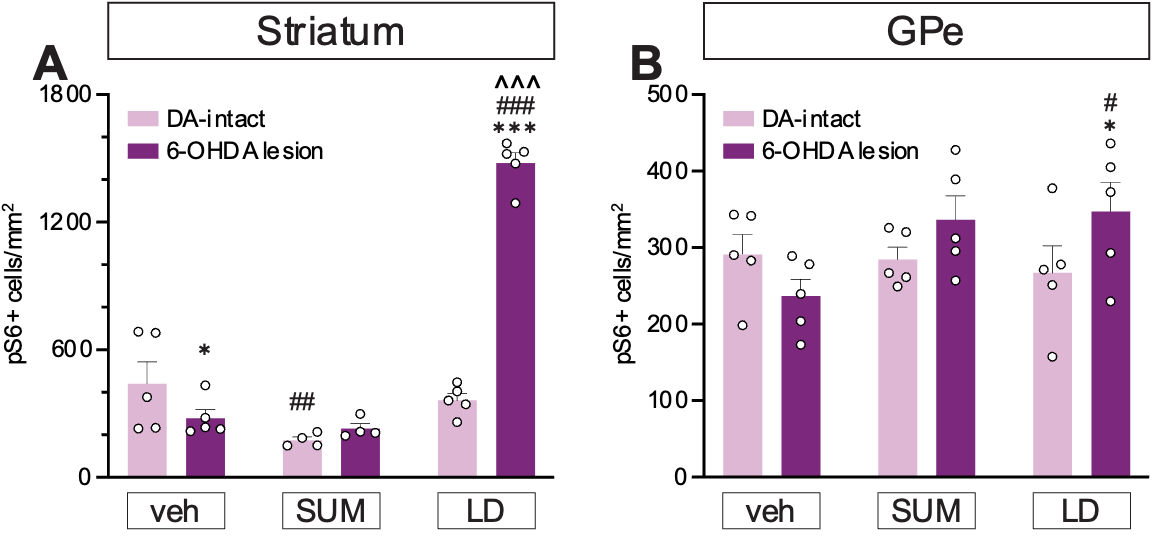
**

**Figure S6. Comparison of pS6-positive cell numbers between intact and DA-denervated sides.** These data were obtained from the unilaterally 6-OHDA-lesioned animals with intact D2R signaling represented in Figure 4. The results show that treatment with L-DOPA or the D2R agonist sumanirole significantly increased the number of pS6-positive neurons in the dorsal striatum and the GPe on the DA-denervated (purple bars) but not the intact hemisphere (pink bars). *Abbreviations:* Veh, vehicle (saline); SUM, sumanirole; LD, L-DOPA. **(A)** *Two-way ANOVA: Lesion: F_(1, 11)_ 93.70, p < 0.001; Treatment: F_(2, 11)_ 64.69, p < 0.001; Interaction: F_(2, 11)_ 137.6, p < 0.0001.* **(B)** *Two-way ANOVA: Lesion: F_(1, 12)_ 2.01, p = 0.1815; Treatment: F_(2, 12)_ 1.13, p = 0.3561; Interaction: F_(2, 12)_ 5.08, p < 0.05. Data is represented as Mean ± SEM. Tukey’s post hoc comparisons. p* < 0.05, *** < 0.001 (intact vs lesion for same treatment) p## < 0.01, ### < 0.001(vs veh for same hemisphere) p^^^ < 0.001(vs SUM for same hemisphere)*

**Table S1. Detailed statistical information for data presented in Figures 2-5***Abbreviations: RM, repeated measures.*

| **Figure 2** | **Test** | **Test statistics** | **p-value** |
| --- | --- | --- | --- |
| **A** | Two-way RM ANOVA | Time: F_(2.0, 40.5)_ 63.13, p < 0.001; Genotype: F_(2, 20)_ 4.19, p < 0.05; Interaction: F_(12, 120)_ 1.63 | 0.093 |
| **B** | Kruskal-Wallis test | KW(genotype) = 6.65 | <0.05 |
| **C** | Two-way RM ANOVA | Genotype: F_(2, 20)_ 4.19, p < 0.05; AIM subtype: F_(1.3, 26.9)_ 13.99, p < 0.001; Interaction: F_(4, 40)_ 1.24 | 0.3084 |
| **D** | Two-way RM ANOVA | Behavior: F_(1.1, 21.4)_ 76.47, P<0.001; Genotype: F_(2, 20)_ 0.99, P=0.3875; Interaction: F_(2.1, 21.4)_ 0.36, P= 0.7138. | 0.7138 |
| **E** | Two-way RM ANOVA | Time: F_(3.6, 71.8)_ 26.23, p < 0.001; Genotype: F_(2, 20)_ 0.46, p = 0.635; Interaction: F_(12, 120)_ 1.44 | 0.1566 |
| **F** | Kruskal-Wallis test | KW(genotype) = 0.83 | 0.6607 |
| **G** | Kruskal-Wallis test | KW(genotype) = 4.74 | 0.0932 |
| **H** | Two-way RM ANOVA | Genotype: F_(2, 20)_ 3.66, p < 0.05; Dystonia subtype: F_(1.8, 35.1)_ 35.51, p < 0.001; Interaction: F_(3.5, 31.1)_ 2.91 | <0.05 |
| **I** | Two-way RM ANOVA | Time: F_(2.4, 47.8)_ 27.09, p < 0.001; Genotype: F_(2, 20)_ 36.96, p < 0.001; Interaction: F_(14, 140)_ 10.49 | <0.001 |
| **J** | Kruskal-Wallis test | KW(genotype) = 19.19 | <0.001 |
| **K** | Two-way RM ANOVA | Genotype: F_(2, 20)_ 36.96, p < 0.001; AIM subtype: F_(1.0, 20.4)_ 98.35, p < 0.001; Interaction: F_(4, 40)_ 36.67 | <0.001 |
| **L** | Two-way RM ANOVA | Behavior: F_(1, 21)_ 92.98, P < 0.001; Genotype: F_(2, 20)_ 15.46, p < 0.001; Interaction: F_(2, 21)_ 17.59 | <0.001 |
| **M** | Two-way RM ANOVA | Time: F_(4.5, 98.3)_ 27.93, p < 0.001; Genotype: F_(2, 22)_ 15.27, p < 0.001; Interaction: F_(8.9, 98.3)_ 5.53 | <0.001 |
| **N** | Kruskal-Wallis test | KW(genotype) = 14.98 | <0.001 |
| **O** | Kruskal-Wallis test | KW(genotype) = 16.76 | <0.001 |
| **P** | Two-way RM ANOVA | Genotype: F_(2, 20)_ 31.21, p < 0.001; dystonia subtype: F_(2.3, 45.9)_ 28.08, p < 0.001; Interaction: F_(4.6, 45.9)_ 22.57 | <0.001 |
| **Figure 3** | **Test** | **Test statistics** | **p-value** |
| **A** | Two-way RM ANOVA | Genotype: F_(2.2, 42.3)_ 136.1, p < 0.001; Genotype: F_(1, 19)_ 43.95 p < 0.001; Interaction: F_(8, 152)_ 13.81 | <0.001 |
| **B** | Mann-Whitney U test |  | <0.001 |
| **C** | Two-way RM ANOVA | Genotype: F_(1, 19)_ 43.95, p<0.001; AIM subtype: F_(1.3, 24.7)_ 33.16, p < 0.001; Interaction: F_(2, 38)_ 14.89, | <0.001 |
| **D** | Two-way RM ANOVA | Behavior: F_(1.3, 24.0)_ 16.70, p < 0.001; Genotype: F_(1, 18)_ 2.27, P = 0.1495; Interaction: F_(1.3, 24.0)_ 5.97 | <0.05 |
| **E** | Two-way RM ANOVA | Time: F_(3.2, 60.7)_ 25.39, p < 0.001; Genotype: F_(1, 19)_ 27.88, p < 0.001; Interaction: F_(3.2, 60.7)_ 10.75 | <0.001 |
| **F** | Mann-Whitney U test |  | <0.001 |
| **G** | Mann-Whitney U test |  | <0.001 |
| **H** | Two-way RM ANOVA | Genotype: F_(1, 19)_ 22.49, p < 0.001; Dystonia subtype: F_(1.6, 31.2)_ 42.00, p < 0.001; Interaction: F_(3, 57)_ 28.52 | <0.001 |
| **I** | Two-way RM ANOVA | Time: F_(2.7, 51.2)_ 30.70, p<0.001; Genotype: F_(1, 19)_ 100.70, p<0.001; Interaction: F_(7, 133)_ 30.70 | <0.001 |
| **J** | Mann-Whitney U test |  | <0.001 |
| **K** | Two-way RM ANOVA | Genotype: F_(1, 19)_ 100.70, p < 0.001; AIM subtype: F_(1.2, 22.0)_ 136.60, p < 0.001; Interaction: F_(2, 38)_ 136.6 | <0.001 |
| **L** | Two-way RM ANOVA | Behavior: F_(1.9, 37.0)_ 15.45, P < 0.001; Genotype: F_(1, 19)_ 9.78, P < 0.01; Interaction: F_(1.9, 37.0)_ 24.20 | <0.001 |
| **M** | Two-way RM ANOVA | Time: F_(4.0, 76.4)_ 37.39, p < 0.001; Genotype: F_(1, 19)_ 223.60, p < 0.001; Interaction: F_(4.0, 76.4)_ 36.91 | <0.001 |
| **N** | Mann-Whitney U test |  | <0.001 |
| **O** | Mann-Whitney U test |  | <0.001 |
| **P** | Two-way RM ANOVA | Genotype: F_(1, 19)_ 37.28, p < 0.001; Dystonia subtype: F_(2.0, 37.2)_ 32.20, p < 0.001; Interaction: F_(2.0, 37.2)_ 36.55 | <0.001 |
| **Figure 4** | **Test** | **Test statistics** | **p-value** |
| **A** | One-way ANOVA | F(treatment)_2,7.39_ =127.1 | <0.001 |
| **B** | Two-way ANOVA | Cell type: F_(1, 11)_ 374.4, P < 0.001; Treatment: F_(2, 11)_ 13.80, P < 0.01; Interaction: F_(2, 11)_ 18.07 | <0.001 |
| **C** | One-way ANOVA | F(treatment)_2,11.45_ =6.11 | <0.05 |
| **D** | Two-way ANOVA | Cell type: F_(1, 12)_ 212.8, P < 0.001; Treatment: F_(2, 12)_ 1.30, P = 0.3070; Interaction: F_(2, 12)_ 1.01 | 0.3903 |
| **Figure 5** | **Test** | **Test statistics** | **p-value** |
| **A** | Two-way ANOVA | Treatment: F_(2, 49)_ 617.0, p < 0.001; Genotype F_(2, 49)_ 0.36, p = 0.7020; Interaction: F_(4, 49)_ 1.84 | 0.1362 |
| **B** | Two-way ANOVA | Treatment: F_(2, 46)_ 122.1, p < 0.001; Genotype F_(1, 46)_ 6.49, p < 0.05; Interaction: F_(2, 46)_ 10.16 | <0.001 |
| **C** | Two-way ANOVA | Treatment: F_(2, 48)_ 21.30, p < 0.001; Genotype F_(2, 48)_ 11.56, p < 0.001; Interaction: F_(4, 48)_ 5.65 | <0.001 |
| **D** | Two-way ANOVA | Treatment: F_(2, 44)_ 7.65, P < 0.01; Genotype F_(1, 44)_ 8.41, P < 0.01; Interaction: F_(2, 44)_ 7.07 | <0.01 |
|  |  |  |  |
